# Moral Convictions and Meat Consumption—A Comparative Study of the Animal Ethics Orientations of Consumers of Pork in Denmark, Germany, and Sweden

**DOI:** 10.3390/ani11020329

**Published:** 2021-01-28

**Authors:** Thomas B. Lund, Sigrid Denver, Jonas Nordström, Tove Christensen, Peter Sandøe

**Affiliations:** 1 Department of Food and Resource Economics, University of Copenhagen, 1958 Frederiksberg C, Denmark; sd@ifro.ku.dk (S.D.); jno@ifro.ku.dk (J.N.); tove@ifro.ku.dk (T.C.); pes@sund.ku.dk (P.S.); 2 Department of Veterinary and Animal Sciences, University of Copenhagen, 1870 Frederiksberg C, Denmark

**Keywords:** welfare-enhanced meat, animal ethics, meat consumption, market-driven animal welfare improvements, cross-cultural comparison, consumer segmentation

## Abstract

**Simple Summary:**

In western Europe, national animal welfare legislation since the 1980s in combination with EU legislation has served to ensure minimal requirements for the welfare of farm animals. For many consumers, however, these requirements do not go far enough. Market-driven initiatives where farmers, processors of animal products, and retailers raise the standards via labelling schemes and price premiums may further improve the welfare of farm animals, but such initiatives are only viable solutions if there is sufficient consumer support. To find out to what extent such support exists, we studied the relationship between animal ethics orientations and consumer demand for welfare-enhanced pork in Denmark, Germany, and Sweden. In all three countries, we identified a consumer segment that endorses the ideal behind schemes to enhance farm animal welfare, i.e., that it is ethically justified to eat meat provided the animals enjoy a good level of welfare. Consumers in this segment are highly concerned about animal welfare, and also purchase welfare pork more often than other consumers. More than one fourth of consumers in all three countries belong to this segment; therefore, we believe that market actors can be reassured that there will be persistent consumer demand for welfare-enhanced meat.

**Abstract:**

Background: The relationship between animal ethics orientations and consumer demand for meat with high standards of animal welfare, and the way this relationship plays out in different countries, is not well understood. Using pork as a case study, this comparative study aims to identify the animal ethics orientations that drive purchases of welfare meat in Denmark, Germany, and Sweden. Methods: Cross-sectional questionnaire data from representative samples of approximately 1600 consumers in each country were collected. A segmentation of pork consumers (using latent profile analysis) was carried out. Results: In all three countries, two subgroups were concerned about farm animal welfare: the first subgroup was driven by animal rights values; the second subgroup by animal protection values, where the main principle was that “it is all right to use animals as long as they are treated well”. Other consumer groups are less concerned about farm animal welfare and display little or no preference for welfare pork. Conclusions: In all three countries, dual demand for welfare pork exists. The findings of this study can be used, among others, to understand the marketability of enhanced welfare animal products and the potential for market-driven animal welfare improvements.

## 1. Introduction

The intensification of animal production has led to affordable meat and other animal products in large parts of the world. However, since at least the 1960s, in many Western countries, concern has been growing about the welfare of hyper-productive farm animals kept in various forms of confinement with little opportunity to engage in natural behavior. In some parts of the world, notably in western Europe, this concern has led to the introduction of legislation intended to protect animals [1], including farm animals [2,3].

For many consumers, the animal protection standards enshrined in current legislation do not go far enough. The majority of the population in many European countries support improvements being made to the welfare of farm animals that go beyond the current legal minimum [4]. Such improvements could be secured by enshrining higher minimum standards in law. However, in many cases, legislation will lead to significant extra costs per unit produced, and in a highly competitive international market for meat and other animal products, this may lead to a dilemma between, on the one hand, maintaining competitive animal production and, on the other, maintaining national legislation that imposes high animal welfare standards.

This dilemma has limited the introduction of new legislation aimed at securing major improvements in the welfare of farmed animals [5]. However, such improvements can also be, and are being, developed voluntarily by market actors [6,7]. Farmers, processors of animal products, and retailers are increasingly raising standards of farm animal welfare to meet consumer demand as people become critical of the conditions on farms operating merely to minimum standards. The welfare-enhanced animal products and potentially interested consumers can be connected through product labeling [8].

### 1.1. Animal Ethics Orientations and Welfare-Enhanced Meat

It is well-documented that many vegetarians and vegans are motivated by their beliefs about animal rights [9,10,11]. Furthermore, it is also known that concern about animal welfare can motivate people to purchase meat and other animal products that have been produced with extra concern for animal welfare [8]. However, the specific animal ethics viewpoints and arguments driving the consumption of welfare products have not received much scholarly attention to date. The notion of an animal ethics orientation can be defined as an underlying set of ethical beliefs about how we—human beings—should treat animals [12,13]. Such orientations shape people’s opinions about, and behavior toward, animals [14,15].

Investigations of the attitudinal and moral drivers associated with a preference for welfare meat have already looked at the following factors: general moral views [16]; personal values [17]; environmental awareness [16]; perceptions of, and opinions about, farmers and the conditions in which farm animals are managed [18,19]; attitudes toward production systems [20]; and concern about animal welfare [8,21]. Other research has examined the willingness-to-pay for welfare meat [22] and the effect of information on consumer preferences for animal food production methods [23]. 

Studies of animal ethics orientations could be an important addition because such orientations may be attitudinal precursors that shape how people think and form opinions about farm animal welfare and, in the end, whether there is a preference for and willingness to purchase welfare products.

The attitudinal measures related to animal use developed (mainly) in the 1990s and 2000s did not include distinct animal ethics orientations [24,25,26]. Contemporary measures have been developed [10,14,15] that allow the empirical identification of the multiple animal ethics orientations that are assumed to co-exist in Western societies [12,13]. The orientations identified in the studies comprise a combination of “pure” animal ethics theories and lay (or intuitive) ethical orientations that are likely to be prevalent in modern societies. 

There are overlaps in the multi-dimensional measures developed. Here, we briefly highlight the orientations identified in the measure presented by Lund et al. [14], because it is used in the present study. Lund et al. [14] identified an animal rights orientation, the central idea of which is that animals matter in the same way as humans do because they are all sentient beings. Consequently, animals should not be used and sacrificed for the sake of humans. In contrast, the anthropocentric orientation assumes that human beings are the center of the moral universe, and that animals are a means of satisfying human needs and interests. Animal protection is characterized by the principle that it is acceptable for humans to use animals, as long as they are treated well and do not experience pain, stress or other forms of suffering. Finally, the lay utilitarian orientation takes its point of departure in the weighting of the utility derived from the use of animals and the harm that animals suffer as a result. It primarily relates to extreme trade-offs between animal pain and human benefits (e.g., the use of animals in bio-medical research), and uses that would ordinarily be regarded as morally contentious. 

### 1.2. What are the Animal Ethical Drivers of Welfare-Enhanced Meat Consumption?

We can think of two animal ethical drivers of welfare-enhanced meat consumption. Firstly, because concern for animal rights is an important motivation for vegetarianism [9,10,11], it could similarly motivate the purchase and consumption of welfare-enhanced meat. On the other hand, perhaps consumers who prioritize animal rights will reject meat consumption and, therefore, welfare-enhanced production facilities, because the use of animals for meat constitutes a violation of animal rights. 

The animal protection orientation only accepts the use of animals if they are treated well; therefore, this orientation could also be a moral driver that prompts consumers to purchase animal welfare meat. However, the only study that has examined this to date found that the animal protection orientation was negatively associated with the consumption of welfare-friendly meat [14]. The above-mentioned study had some limitations, however. Firstly, the link between animal ethics orientations and the consumption of welfare meat was examined using only one question. Therefore, it lacked detail considering the many meat variants that are available in stores. Secondly, the assessment was only made on one population (Danish consumers), which means that the generalizability of the identified association is limited. A third limitation was that Lund et al. [14] employed population-wide correlational analyses to study the association. However, correlational analysis does not reveal possible consumer heterogeneity very well. 

### 1.3. Segmenting Consumers on the Basis of Animal Ethics Orientations and Attitude Strength

Cluster analysis divides populations into subgroups (or segments). In this study, cluster analysis was used to study consumer heterogeneity in animal ethics orientations. In particular, we used cluster analysis to identify consumer segments on the basis of the following animal ethics orientations: animal rights, animal protection, and anthropocentrism (we omitted the lay utilitarian orientation because it primarily relates to extreme trade-offs between animal pain and human benefits [14]). Potentially, a cluster analysis identifies segments of consumers with a distinct set of animal ethics orientations and concurrent propensity to purchase welfare meat. 

As part of this clustering of consumers, we believe that it is also advantageous to differentiate within consumer concern for farm animal welfare. This can be empirically addressed by focusing on attitude strength. Attitude strength is a concept developed by attitude theorists [27] to reflect the fact that the strength of a particular attitude varies between individuals. The stronger the attitude, the more it shapes an individual’s mind-set, their considerations in relevant situations, and ultimately their behavior, which includes product choice [28]. It has been shown that attitudes primarily determine behavior in individuals with high attitude strength [28,29]. 

In the cluster analysis, we included a measure of attitude strength that captures two of its features: importance, which is the extent to which an individual considers the issue to be important; and accessibility, which is the extent to which an attitude is activated in relevant situations [27], in this case, the purchase situation. Our aim with this was to identify segments with high attitude strength so that their animal ethics orientations could be determined. While conceptually dissimilar, attitude strength can be viewed as (possibly) closely related to purchase behavior. Therefore, we assumed that segments that are concerned about animal welfare purchase welfare-enhanced products more frequently. However, in order to ensure that this is, in fact, the case, we also examined this assumption. 

### 1.4. National Variation

Animal ethics theorists assume that animal ethics orientations are universal and prevail across countries [13]. However, the orientations may be differentially linked with consumers’ choice of meat because the production of meat and the surrounding welfare legislation varies between countries. Countries also differ in terms of the interaction that occurs between private actors (e.g., retailers and producers) and public policies [2,7]. Therefore, the link between animal ethics orientations and welfare meat consumption may vary between cultures.

For this reason, the present study compared the animal ethics orientations that drive purchases of welfare meat across countries. The investigation was based on questionnaire data on consumers from three countries: Denmark, Germany, and Sweden, and focused on a single type of meat: pork. The aim was to obtain a detailed insight into the purchase patterns for a range of products from different production schemes, including conventional pork and pork from pigs with enhanced welfare.

The study countries made it possible to evaluate whether different background conditions affect the link between animal ethics orientations and meat consumption, because the balance between maintaining internationally competitive animal production and imposing animal welfare legislation is different in the three countries:

Sweden has introduced animal welfare legislation that gives pigs and other farm animals a level of protection that goes well beyond the minimum requirements laid down in EU law. Consequently, its pork production mainly serves the domestic market. In Swedish shops, there is a combination of domestically produced pork, with enhanced welfare, and cheaper imported pork, typically produced to lower welfare standards than those found in Sweden. A small share of Swedish pork is organically produced, and here the welfare standards are higher than those found in conventional domestic production [30]. 

In Denmark and Germany, on the other hand, animal welfare legislation covering pork and other kinds of animal production does not extend far beyond the minimum requirements set by the EU [30]. However, as a result of the desire to cater for the needs of consumers who are concerned about farm animals, the market for welfare products has developed substantially over the last decade. In the case of welfare pork, two levels are currently available in both countries: premium, i.e., organic and other forms of production with outdoor piglet production and access to outdoor areas for the remaining pigs; and enhanced indoor, i.e., production in indoor facilities but without tail docking, with 100% loose-housed sows, and extra provision of space and straw for slaughter pigs, among other things [6]. In Denmark, the two forms of production have been supported by a government-run labeling system. Furthermore, a major Danish animal welfare NGO and a large food retailer have cooperated to develop an alternative label that is available in supermarkets run by the retailer. In Germany, the labeling system for enhanced indoor pork has been developed by market actors, including producers and retailers [30]. 

### 1.5. Research Aims

The overall aim of this comparative study was to identify the animal ethics orientations (if any) that drive purchases of welfare meat in the three countries using pork as a case study. The analysis progressed as follows: first, we compared the prevalence of the animal ethics orientations in the three countries; secondly, we used latent profile analysis (a cluster analysis method) to identify consumer segments with different animal ethics orientations and levels of attitude strength; thirdly, we analyzed the pork purchase pattern of the identified consumer segments.

## 2. Materials and Methods

### 2.1. Data

The study used cross-sectional online questionnaire data from Denmark, Germany, and Sweden, which was collected by a survey company (Norstat, Denmark; Norstat, Germany; Norstat, Sweden) that hosts and maintains a panel of consumers in all three countries. For each country-specific panel, a stratified random gross sample of members of the panel was drawn, after which data were collected until approximately 1600 respondents per country had completed the questionnaire (stratification variables were gender, age, and region). The number of individuals invited to respond to the questionnaire in Denmark, Germany and Sweden were 9099, 5680 and 6450, respectively, of whom 1612, 1607 and 1613 completed the questionnaire, giving response rates of 18%, 28% and 25%. The data were collected in June 2019. Socio-demographic details of the country-specific samples are reported in Table 1.

### 2.2. Compliance with Ethical Standards

The study included human participants that responded to a questionnaire. Informed consent complying with Danish legislation was collected and handled by the survey bureau (Norstat). The researchers received the data from the bureau in a pseudonymized form so that no personal identifiable information was disclosed. The study was approved by the Institutional Review Board at the Faculties of Science and of Health and Medical Sciences at the University of Copenhagen (journal no.: 504/0159/20-5000). 

### 2.3. The Questionnaire

We strove to provide the same questions and response options to respondents in all countries. However, some country-specific differences existed, such as the types and labels of pork products on the market, which meant that some differences were unavoidable. The questionnaire was written in Danish by the authors. The survey company then translated it into German and Swedish. The authors (who have good knowledge of German and Swedish) checked the translations and, after a few rounds of corrections, a final version of the questionnaire was produced. This was piloted in all three countries (*n* = 200 in each country). A few further alterations were made following this pilot. Data from the piloting of the survey were not included in the study. 

### 2.4. Measures

Animal ethics orientations: following Lund et al. [14] we used 12 statements to measure the four animal ethics orientations that are discussed in the introduction: animal rights, anthropocentrism, animal protection, and lay utilitarianism. Response options ranged from 1: “completely disagree” to 5: “completely agree” (See Supplementary File S1 for an overview of the language-specific versions of the 12 statements). To ensure that the hypothesized ethics structure involving the four orientations was similar across the three countries, we tested for measurement invariance [31], the details of which can be found in Supplementary File S2. The conclusion was that the ethics structure is similar between the countries, which means it is statistically acceptable to compare mean scores between countries.

In line with the study by Lund et al. [14], four composite variables were constructed, one for each of the orientations. The raw scores of the factor-specific items were summed (see equation in the notes to Table 2), and rescaled to range between 0 and 100, where 0 indicates a very low, 100 a very high, and 50 a medium propensity to subscribe to the animal ethics orientation in question. 

Attitude strength: this measure, which is based on four items, captures two features of attitude strength: importance and accessibility [27]. There were two attitude importance items: “In my view, all the talk about animal welfare is exaggerated” and “Society has more important things to focus on than the condition of animals”. Response options were: (a) “totally disagree”; (b) “disagree”; (c) “neither agree nor disagree”; (d) “agree”; (e) “totally agree”; and (f) “don’t know”. We took disagreement with the statements to indicate attitude importance and recoded responses to the two items into the values 1 = disagreement (responses a, b), or agreement = 0 (responses c, d, e, f).

There were two attitude accessibility items: “When I purchase pork, I think about how the pigs were treated” and “I can’t be bothered to familiarize myself with the requirements of different animal welfare labels”, both of which had the same five-point response options as the attitude importance items. We took agreement with the first item (binary recoded into: agreement = 1 (responses d, e) or agreement = 0 (responses a, b, c, f), and disagreement with the second item (binary recoded into disagreement = 1 (responses a, b) or 0 (responses c, d, e, f) to indicate attitude accessibility. 

In all three countries, the four items exhibited acceptable internal consistency as the ordinal alpha coefficient — which is the recommended internal consistency coefficient for binary items [32]—was well above the commonly agreed upon acceptance threshold of 0.70 (ordinal alpha: Denmark = 0.81; Germany = 0.76; Sweden = 0.79). We calculated a composite score by summing the responses to the five items, which gave a measure of attitude strength with the score range of 0 to 4 with higher scores indicating higher attitude strength. 

Purchase of pork: the respondents were asked to report the frequency with which they purchase country-specific pork types (response options: “never”, “seldom”, “sometimes”, “often”, “almost every time I buy pork” and “I don’t know”). In all countries, respondents were prompted about conventional pork. In Germany and Denmark, they were prompted about two types of welfare pork: type 1 and type 2. Type 2 pork has the highest welfare standards and involves outdoor rearing. This is typically, but not always, organic meat (German question: “Bio-Schweinefleisch (z. B. Naturland)”; Danish question: “Svinekød fra udendørs produktion mærket med fx Økologisk Svinekød, Friland Svinekød eller ‘Anbefalet af Dyrenes Beskyttelse’”). Type 1 pork involves some welfare improvements above minimum standards and is typically, but not always, from indoor production (German question: “Schweinefleisch mit Labels wie Initiative Tierwohl, Haltungsform, Für Mehr Tierschutz, Tierschutz kontrolliert oder Neuland”; Danish question: “Svinekød fra indendørs produktion mærket med fx Fødevarestyrelsens velfærdsmærke ‘Bedre dyrevelfærd’, COOP’s dyrevelfærdshjerte eller Antonius”). In Sweden, where legislation affords pigs and other farm animals a level of protection that roughly equals that found in type 1 pork in Germany and Denmark, a market for medium level welfare pork has not emerged. Therefore, in this case we only enquired about one type of welfare pork, corresponding to type 2 pork (“Ekologiskt fläskköt”).

### 2.5. Data Analysis

Differences in mean scores for the four animal ethics orientations in the three countries were examined using analysis of variance. For each country, correlations between the orientations and with socio-demographic factors were reported.

To identify consumer segments, we conducted latent profile analysis (LPA) [33], which has the advantage that it can take into account the measurement error that occurs when individuals are assigned to clusters [34]. We inserted the following four variables in the LPA: animal rights, anthropocentrism, animal protection, and attitude strength. We treated all four input variables as continuous. Because the attitude strength variable had kurtosis issues (kurtosis < −1 in all countries), we checked the robustness of the results by running the analysis in which the attitude strength variable was treated as ordinal. This did not change the pattern of the results; therefore, we chose to treat the variable as continuous to simplify the presentation. 

When choosing the number of clusters (this will also be referred to as classes in the subsequent text) to report, we relied on a combination of fit indices and conceptual considerations about the relevance of the latent classes given our research aims [35]. The conceptual considerations were important because a central aim was to identify the animal ethics profiles of segments that are concerned about animal welfare. We examined four statistical fit indices when evaluating the number of classes: the Akaike Information Criterion (AIC [36]), and the sample-size adjusted Bayesian Information Criterion (BIC [37]) (for AIC and BIC, lower values reflect better fitting latent class models), the Lo–Mendel–Rubin Likelihood Ratio Test (LMR-LRT [38]) (for which a probability value *p* < 0.05 indicates that the model with x classes provides a significantly better fit to the data than the model with x−1 classes), and entropy [39]. The LPA model search results are reported in Supplementary File S3. Following a model search with up to six classes, the fit statistics did not agree regarding the optimal number of classes within or between countries. An inspection of the four-class model in all the countries revealed a division into two animal welfare-concerned segments with high levels of attitude strength. This division was not apparent in the two-class and three-class models. The addition of five or six classes did not provide further relevant divisions into welfare-concerned consumer segments. Therefore, we chose to proceed with the four-class model.

Gender, age, and income characteristics of, and tests of difference between, the consumer segments (i.e., latent classes identified through LPA) were calculated separately for each country using the DCAT option (for categorical distal outcomes) and DCON option (for continuous distal outcomes) implemented in Mplus [40]. Purchase frequencies of the three (and in Sweden, two) pork types in each consumer segment were also calculated using the DCAT option (for categorical distal outcomes) [40]. The DCAT (and DCON) approach accounts for the estimation error that occurs when assigning individuals to latent classes [34]. Unadjusted and adjusted tests of difference between the consumer segments in terms of purchase frequencies of the pork types were estimated using the manual BCH distal outcome method [40]. The adjusted test statistic was carried out with the aim of investigating whether differences between the classes in pork consumption remained after having controlled for other possible explanatory factors. The following fourteen variables were included as control variables in the adjusted analysis: three socio-demographic factors (gender, age and income) and eleven binary variables indicating the most important considerations when purchasing pork (based on the question “What is important when buying pork? Choose up to five things”): ease of purchase, appropriate pieces/cuts, domestically produced, taste, inexpensive, low fat, low environmental burden, low climate burden, food safety, no use of antibiotics, and no use of genetically modified feedstuff.

Post-stratification weights were calculated that adjusted for under- or overrepresentation of socio-demographic strata in the sample relative to national census data. The following strata were adjusted for: gender, age, region, and education. The post-stratification weights were applied in all descriptive analyses. 

Statistical software used included SPSS Statistics version 27: Armonk, NY: IBM Corp (used predominantly for data management and descriptive statistics,), and Mplus Version 8.4: Los Angeles, CA: Múthen and Múthen (used for the latent profile analysis and analysis of measurement invariance). 

## 3. Results

### 3.1. Country Differences in Animal Ethics Orientations

In Table 2, average scores for the four orientations are reported for each country along with the percentage of respondents who agreed with the 12 questions, which was used to construct the four variables that indicated the orientations. Statistically significant differences exist between the countries for all four orientations (in all cases: *p* < 0.001). The average Danish consumer is more animal protection oriented (score 69.5), anthropocentrically oriented (47.0) and lay utilitarian oriented (38.3) than the average German consumer (animal protection = 57.7; anthropocentric = 40.3; lay utilitarian = 28.8), and the average Swedish consumer (animal protection = 65.5; anthropocentric = 42.1, lay utilitarian = 34.5). The average German consumer is more animal rights oriented (score 47.6) than the average Danish (40.8), and Swedish (40.4) consumer. 

However, overall, the differences across countries regarding overall prevalence levels are not particularly marked and the general patterns are notably similar between the three countries: the lay utilitarian orientation is least prevalent (scores between 28.7 (Germany) and 38.3 (Denmark)). The animal protection orientation is most prevalent (scores between 57.7 (Germany) and 69.9 (Denmark)). The animal rights and anthropocentric orientations are positioned between these two, with average scores in the 40–50 region across countries.

In all of the study countries, the animal rights position is negatively correlated with the three other orientations, while the remaining three orientations are positively correlated (Table 3). There is some difference in the strength of the correlation, particularly between animal rights and animal protection (minimum (Germany) = −0.346; maximum (Denmark) = −0.547), but the country differences are, in general, modest. 

The associations between orientations and socio-demographic factors are quite similar across the countries. There is no, or only a modest, correlation, with age. Women are more animal rights oriented (Spearman’s r between 0.209 and 0.269), while men are more strongly oriented to anthropocentricism (Spearman’s r between −0.170 and −0.283), animal protection (Spearman’s r between −0.109 and −0.205) and lay utilitarianism (Spearman’s r between −0.268 and −0.310). The animal rights orientation decreases, while the anthropocentric, animal protection and lay utilitarian orientations increase with higher household income.

### 3.2. Segmenting Pork Consumers by Animal Ethics Orientation and Attitude Strength

We now move from the population-wide level of analysis to the division of consumers into segments (using LPA) on the basis of their animal ethics orientations and attitudinal strength. 

The results of the four-class solution in the three countries are reported in Table 4. Similar information relating to consumers who were not included in the LPA (those who do not eat or purchase pork, and vegetarians and vegans) is included in the table for comparison.

In all three countries, a rather similar latent class pattern emerges. Compared with population averages, the consumers in latent class 1 have very high anthropocentric and animal protection scores, and a very low animal rights score. They also have limited concern for animal welfare; attitude strength scores are low compared with class 3 and class 4. Consumers in latent class 2 are characterized by an indistinct combination of animal ethics in that they do not ascribe to or reject any particular orientation. All orientations are in the middle region (with scores from 45–65). Furthermore, consumers in class 2 are unconcerned about animal welfare, with low attitude strength. Consumers in class 3 and 4, conversely, are characterized by high concern (i.e., high attitude strength scores), but they differ with respect to orientations. The animal protection scores of the consumers in latent class 3 are higher than the population average, while their animal rights scores are relatively low (and close to the population average). Importantly, for class 3, the anthropocentric orientation is lower than the population average and much lower than in class 1 and 2. Consumers in latent class 4 are clearly below the population average with regards to animal protection and anthropocentrism. However, they have very high scores for the animal rights scale.

### 3.3. The Socio-Demographic Characteristics of the Consumer Segments

Table 5 shows the socio-demographic characteristics of the four pork consumer segments. Across countries, we identify statistically significant differences regarding all three socio-demographic variables reported. 

In all three countries, vegetarians and vegans have very low anthropocentric scores, while the animal rights orientation is higher than the population average. 

We note that age differences between the segments are relatively modest, and that no clear pattern emerges. Across countries, consumers in class 1 are more likely, and consumers in class 2 and class 4 are less likely, to be in the highest income quintile. The share of consumers in class 3 in the highest income quintile is close to (or slightly above) the overall population share. Turning to gender, we identify very clear differences between the segments, and the differences are do not vary between countries. Consumers in class 1 are predominantly male (the male share ranges from 69% (Denmark) to 83.1% (Germany)). Consumers in class 4 are, on the other hand, predominantly female (the female share ranges from 72% (Germany) to 80% (Sweden)). The gender differences are less marked in class 2 and class 3. 

### 3.4. Consumer Segments and Pork Purchases

In all three countries, statistically significant differences exist in terms of the frequency with which the different pork types are purchased by the four consumer segments. The differences are also statistically significant after controlling for socio-demographic factors and other important consumer considerations for purchasing pork (see Supplementary File S4 for detailed test results).

**Figure 1:**
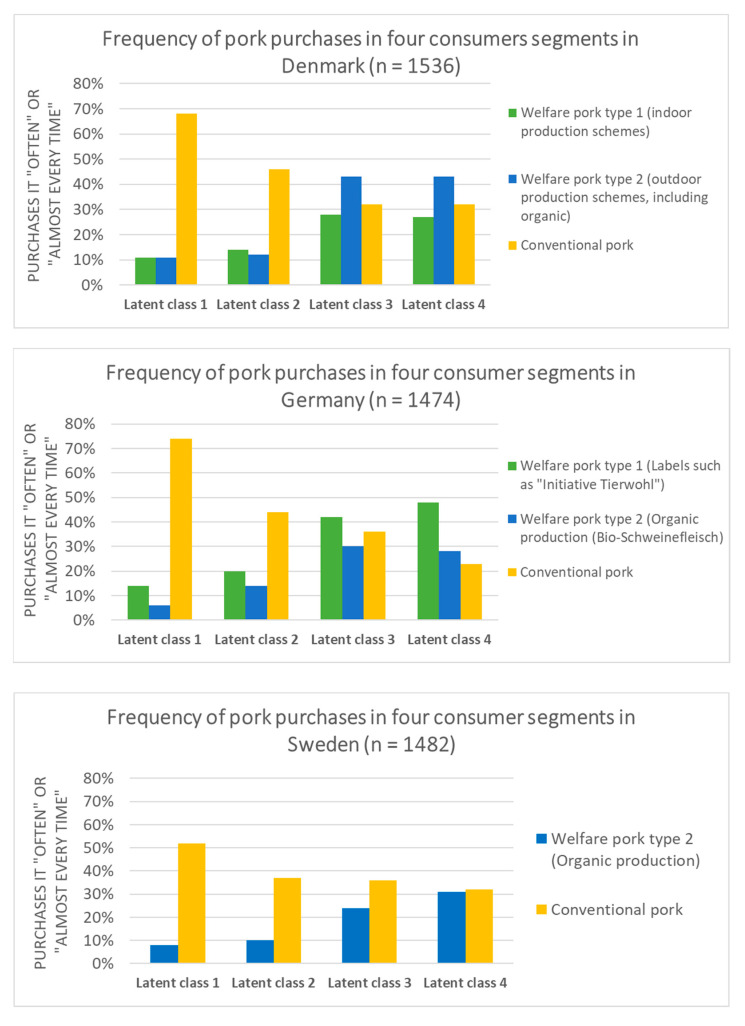
Frequency of purchase of three types of pork in four subpopulations in Denmark, Germany, and Sweden.

The upper part of Figure 1, which presents the results for Denmark, shows that consumers in class 3 and class 4 are more than twice as likely to report that they purchase welfare pork type 1 and 2 “often”/“almost every time” compared with consumers in class 1 and 2. Conversely, consumers in class 1 and 2 in Denmark are much more likely to report that they purchase conventional pork “often”/“almost every time”. 

In Germany (middle part of Figure 1), consumers in class 3 and class 4 are also more likely to report that they eat welfare pork type 1 and welfare pork type 2 “often”/“almost every time” than consumers in class 1 and class 2. Consumers in class 1 are much more likely to report that they purchase conventional pork “often”/“almost every time”. Consumers in class 4 (highly animal rights oriented) are least likely to purchase conventional meat. 

In Sweden (lower part of Figure 1), the differences are less marked than they are in Denmark and Germany. However, clear and statistically significant differences still exist between the consumer segments. Consumers in class 3 and 4 are two to three times more likely to report that they purchase welfare pork type 2 (compared with consumers in class 1 and class 2). Consumers in class 1, who are highly anthropocentric, are more likely to report that they purchase conventional pork “often”/“almost every time” than the other consumer segments. 

## 4. Discussion

In all three countries, two consumer segments with a high propensity to buy welfare pork and a lower propensity to purchase conventional pork were identified (class 3 and class 4). Both displayed high levels of animal welfare concern (high attitude strength), but one of the segments was driven by an animal rights orientation, while the other by an animal protection orientation. This between-country similarity emerged despite the significant differences between the countries regarding welfare legislation and the interaction between private actors (e.g., retailers and producers) and public policies [2,7]. 

This bifurcation in demand has important practical implications for the future prospects for the market-driven promotion of higher standards of animal welfare. Specifically, producers and advocates of market-driven improvements to animal welfare will be reassured that a consumer segment that is willing to accept the ideal behind enhanced farm animal welfare production schemes, i.e., that it is ethically acceptable to eat meat provided the animals enjoy a good level of welfare, exists across the studied countries. This fundamental approach to animal use, which we captured through the animal protection orientation, is widely supported by consumers in class 3, the members of which are also highly concerned about animal welfare (cf. their attitude strength score), and purchase welfare pork more often than members of other consumer segments. A relatively high share of consumers (25.4% (Germany) to 36.9% (Sweden)) belong to this class (however, we note that these shares should be treated with caution because they are dependent on (and can vary as a function of) the specific input variables in the cluster analysis). A major concern for marketers and producers could be that animal welfare-concerned consumers are impeded by practical restriction, e.g., where income (purchasing power) is concerned. Our results do not suggest that purchasing power is a particular challenge for consumers in the relevant class 3. Thus, the share of consumers in class 3 within the highest income quintile is close to (or slightly above) the overall population share. 

The animal protection orientation also has a significant presence in the two consumer segments that do not currently purchase welfare pork very often (class 1 and class 2). Consumers in the two segments would have to start acting on the basis of their animal protection values in order to purchase animal welfare-friendly meat products. Given the level of concern (low attitude strength) of these two consumer segments, at this point in time, it might only be possible to attract their attention through major shifts in the market that make animal welfare meat easier to purchase and affordable compared with the conventional alternatives. This may require initiatives from the retail sector. Retailers, however, are not necessarily concerned about animal welfare [41]. On the other hand, major initiatives taken from the retail sector have been observed. Thus, in the Netherlands, retailers have agreed to only sell fresh chicken meat from broilers that grow more slowly than the standard extremely fast-growing broiler [42]. 

We found that the animal protection orientation is the most prevalent in all countries. Hölker et al. [15] identified a similar orientation, which they labeled the new contractarian approach, and they also found is the most common in Germany [15]. This supports the hypothesis suggested by Garner [13,43], that animal welfarism is widespread and that it serves as a moral justification for eating meat. The so-called “meat paradox” observes that we treat animals very differently: we eat them when they are farm animals, but we also care about them, e.g., when they are pets [44]. To resolve this dissonance, people devise justifications for the continued use of animals by rationalizing the act of eating meat [45], and by decoupling production animals from the association with the brutal handling of animals [46]. In line with this, the reasoning involved in the animal protection discourse (“The use of animals is acceptable if they have had a decent quality of life”) also helps to justify meat consumption. 

The other class of consumers with high demand for welfare-enhanced pork, class 4, which accounts for approximately one-tenth of consumers in the three countries, is predominantly driven by animal rights orientations. It may appear contradictory that consumers in class 4 are willing to eat pork considering their high animal rights orientation. However, these consumers are not exclusively oriented to animal rights: average scores on the animal rights scale ranged from 67 to 72 in the three countries. Therefore, they may be in the process of reducing and eventually phasing out meat consumption. Alternatively, they may be culturally/attitudinally in line with a vegan grouping identified by Janssen et al. [9] who would be tolerant of some types of livestock production if the animal welfare standards were to go beyond current levels. Consequently, consumers in class 4 may have identified animal welfare meat products on the three markets that they consider to be satisfactory and, in turn, may have abandoned the idea of phasing out meat consumption. However, we are unable to examine these speculations in the present dataset.

Across the three countries, the four animal ethics orientations are similarly correlated with each other. Furthermore, all orientations are associated with the same socio-demographic factors: gender and socio-economic status (measured as household income). In the subsequent cluster analysis, we identified major gender differences (again across countries). Women are much more likely to belong to the class of consumers with high animal welfare concern and high animal rights orientation (class 4), while men are much more likely to belong to class 1 where animal welfare concern is low, and anthropocentric orientation high. The pattern of the associations is similar to findings in the U.S. [26] where women and individuals with lower socio-economic status tended to sympathize more with animals and be less open to anthropocentrism and utilitarian calculations. 

Notably, compared with Germany and Sweden, in Denmark the human-centered orientations (animal protection, anthropocentric, and lay utilitarian) were more prevalent. This may be because Denmark has been a large meat exporting country for many years and, thus, Danes have come to see the use of animals for food as natural and essential to their livelihoods. On the other hand, one study found that German consumers are more human centered than Danes [17]. Unfortunately, this study and the present investigation are difficult to compare directly because the animal attitude measures employed are dissimilar and because the two datasets were collected 10 years apart.

The results of the present study also build on and help to clarify the unexpected finding in an earlier study where high animal protection values resulted in a lower propensity to purchase welfare meat [14]. That finding may have been a methodological artifact of the population-wide analysis employed. Thus, at the population level, animal protection scores increase in line with anthropocentric scores (cf. Table 2). The analysis presented in the present study clustered consumers into segments so that multiple animal ethics orientations of relevance could be considered concurrently and in joint consideration with different levels of animal welfare concern (attitude strength). It was this segmentation that enabled us to identify class 3 consumers, who are characterized by high animal protection values, low anthropocentric values, high animal welfare concern, and a higher propensity to purchase welfare meat. 

We note three limitations to the present study. Firstly, stated behavior, such as the frequency of purchasing pork, can result in recall bias. However, because we would expect this bias to be evenly distributed across the sampled participants, the observed difference between consumer segments in reported propensity to purchase welfare and conventional pork is not likely to have been influenced. Secondly, “social desirability bias” may arise because respondents tend to answer questions in ways they think will make them appear favorably before others. Although this bias cannot be ruled out completely, it is well established that the internet survey administration format, where there is no interviewer to impress, is less prone to this bias than interviewer-conducted formats [47]. Thirdly, our response rates were relatively low (between 18% and 25% in the three countries) and only involved a random selection of respondents from pre-recruited panels. However, low response rates are only a limitation if there is non-response bias [48]. Studies suggest that non-response bias is limited (at the level of means and prevalence) when post-stratification weighting is used (which was the case in all of the descriptive results reported in this paper) [49]. 

## 5. Conclusions

The overall aim of this study was to identify the animal ethics orientations (if any) that drive the purchases of welfare meat using pork as a case study in Denmark, Germany, and Sweden. The segmentation of pork consumers identified two subgroups that are concerned about animal welfare in all three countries but have different animal ethics orientations. The first subgroup is driven by animal rights values; the second subgroup by animal protection values where the main principle is that “it is all right to use animals as long as they are treated well”. 

Our research findings offer important insights that may benefit those seeking to analyze the marketability of welfare meat and promote market-driven animal welfare improvements. The study also emphasizes the fact that the heterogeneity of views within populations regarding farm animal welfare issues need to be considered when assessing the market potential of welfare meat. 

Future studies should examine potential trajectories of the unconcerned consumer segments (class 1 and class 2) and identify possible ways of directing these toward the purchase of welfare meat. 

## Figures and Tables

**Table 1:** Socio-demographic details of the three samples (unweighted data).

Socio-Demographic Variables	Denmark (*n* = 1612) Count (%)	Germany (*n* = 1607) Count (%)	Sweden (*n* = 1613) Count (% )
Gender			
Male	791 (49.1)	742 (46.2)	786 (48.7)
Female	821 (50.9)	865 (53.8)	827 (51.3)
Age			
18–33 years	396 (24.6)	283 (17.6)	328 (20.3)
34–49 years	337 (20.9)	454 (28.3)	401 (24.9)
50–64 years	421 (26.1)	567 (35.3)	408 (25.3)
65 or more	458 (28.4)	303 (18.9)	476 (29.5)
Age (in years) Mean (s.e.)	50.5 (0.45)	49.4 (0.40)	48.8 (0.45)
Education ^a^			
Low ^b^	848 (52.6)	848 (52.5)	781 (48.0)
High ^c^	764 (47.4)	763 (47.5)	832 (52.0)
Income ^d^			
Highest quintiles	309 (19.2)	345 (21.5)	339 (21.0)
Other income groups	1303 (80.8)	1262 (78.5)	1274 (89.0)

s.e., standard error. ^a^ Highest completed education. ^b^ Country-specific definitions of low education are Denmark: Folkeskole/Grundskole, Gymnasial uddannelse, Erhvervsuddannelse, Kort videregående uddannelse, and other; Germany: Haupt- (Volks-) schulabschluss, Realschul- oder gleichwertiger Abschluss, and other; Sweden: Grundskola/Realskola, Gymnasium, Folkhögskola, and other. ^c^ Country-specific definitions of high education are Denmark: Mellemlang videregående uddannelse (Bachelor niveau), Lang videregående uddannelse (Kandidat niveau); Germany: Fachhochschul- oder allgemeine Hochschulreife; Sweden: Högskola/Universitet, 60 poäng (40 poäng, gamla systemet) eller mindre, Högskola/Universitet, mer än 60 poäng (40 poäng, gamla systemet). ^d^ Highest quintile in Denmark: > 700,000 DKK/year; Germany: > 3499 EUR/month; Sweden: > 700,000 SEK/year.

**Table 2:** Scores for the four animal ethics scales and share (reported in percent) of consumers that disagree/agree with the specific statements relating to the four orientations in Denmark, Germany, and Sweden.

Questionnaire Items	Denmark (*n* = 1612)			Germany (*n* = 1607)			Sweden (*n* = 1613)		
	Disagree %	Neither/Nor %	Agree %	Disagree %	Neither/Nor %	Agree%	Disagree %	Neither/Nor %	Agree%
Animal rights items									
1. The use of animals by humans should be prohibited by law.	56.8	30.3	12.9	46.2	35.1	18.7	56.3	28.5	15.2
2. In principle, the use of animals by humans is unacceptable because animals can feel pain, happiness, etc.	35.4	38.2	26.4	26.6	37.4	36.1	32.8	35.2	31.9
3. In principle, the use of animals by humans is unacceptable because animals are sentient beings.	41.8	36.4	21.8	30.4	37.3	32.3	36.5	35.7	27.8
Animal rights score									
Mean (s.e.)	40.8 (0.62)			47.6 (0.63)			43.4 (0.65)		
Cronbach’s α	0.89			0.86			0.89		
Anthropocentric items									
4. We have the right to use animals because humans are intellectually superior to animals.	47.4	36.1	16.4	52.0	35.2	12.8	56.7	33.6	9.7
5. Human interests are more important than those of animals.	30.8	37.9	31.3	38.6	39.5	21.9	38.2	36.7	25.1
6. We must prioritize humans over animals.	22.6	40.1	37.3	33.0	41.9	25.2	32.8	39.3	27.9
Anthropocentric score									
Mean (s.e.)	47.0 (0.58)			40.3 (0.58)			40.9 (0.57)		
Cronbach’s α	0.83			0.80			0.83		
Animal protection items									
7. Using animals for important human purposes (e.g., medical research) is acceptable if it is done so that the animals do not experience unnecessary stress.	11.4	23.3	65.3	25.1	32.4	42.6	18.4	27.9	53.7
8. Using animals for important human purposes is acceptable if it is done so that the animals do not experience unnecessary pain.	7.6	21.9	70.5	23.6	31.2	45.2	13.7	24.6	61.8
9. Using animals for important human purposes is acceptable if the animals have a decent quality of life.	7.0	20.4	72.6	14.8	28.0	57.2	12.8	25.2	62.0
Animal protection score									
Mean (s.e.)	69.5 (0.49)			57.7 (0.58)			63.4 (0.57)		
Cronbach’s α	0.82			0.82			0.85		
Lay utilitarian items									
10. Inflicting serious pain on animals is acceptable if it is necessary in order to achieve a vital human goal, e.g., in medical research.	54.1	29.0	16.9	64.1	25.0	10.9	59.8	24.7	15.5
11. Inflicting considerable pain on animals is justified if the purpose is sufficiently important, e.g., medical research.	52.6	28.5	18.9	61.7	27.9	10.4	59.3	26.0	14.7
12. Exposing animals to stress and reducing their welfare is justified if the purpose is sufficiently important.	43.4	35.4	21.1	60.5	28.0	11.5	52.6	29.8	17.7
Lay utilitarian score									
Mean (s.e.)	38.3 (0.63)			28.8 (0.62)			33.5 (0.57)		
Cronbach’s α	0.87			0.92			0.87		

(s.e.): standard error. Scores for the four measures of animal ethics were constructed on the basis of the respondents’ raw responses to the specific items of the orientations, e.g., for the animal rights variable: ((((item1 + item2 + item3)−3/)12)*100). Weighted mean values and shares (agree, neither/nor, and disagree) are reported using post-stratification weights as described in the data analysis section.

**Table 3:** Matrix of correlations between animal ethics orientations, age, gender, and household income reported separately for Denmark, Germany, and Sweden.

**Denmark** (***n* = 1612**)							
**Animan ethics orientation**	Animal Rights	Anthropo-centric	Animal Protection	Lay Utilitarian	Age	Gender (0 = male/1 = female)	Household income (*n* = 1329)
Animal Rights	1.00 ^a^	−0.437 **^,a^	−0.547 **^,a^	−0.342 **^,a^	−0.018 ^a^	0.222 **^,a^	−0.199 **^,a^
Anthropocentric		1.00 ^a^	0.396 **^,a^	0.599 **^,a^	0.061 *^,a^	−0.170 **^,a^	0.201 **^,a^
Animal Protection			1.00 ^a^	0.335 **^,a^	−0.010 ^a^	−0.109 **^,a^	0.178 **^,a^
Lay Utilitarian				1.00 ^a^	−0.076 **^,a^	−0286 **	0.170 **^,a^
**Germany** (***n* = 1607**)							
**Animan ethics orientation**	Animal Rights	Anthropo-centric	Animal Protection	Lay Utilitarian	Age	Gender (0 = male/1 = female)	Household income (*n* = 1430)
Animal Rights	1.00 ^a^	−0.333 **^,a^	−0.346 **^,a^	−0.209 **^,a^	−0.057 *^,a^	0.209 **^,a^	−0.121 **^,a^
Anthropocentric		1.00 ^a^	0.368 **^,a^	0.665 **^,a^	0.018 ^a^	−0.225 **^,a^	0.108 **^,a^
Animal Protection			1.00 ^a^	0.330 **^,a^	0.041 ^a^	−0.167 **^,a^	0.099 **^,a^
Lay Utilitarian				1.00 ^a^	−0.119 **^,a^	−0.268 **^,a^	0.086 **^,a^
**Sweden** (***n* = 1613**)							
**Animan ethics orientation**	Animal Rights	Anthropo-centric	Animal Protection	Lay Utilitarian	Age	Gender (0 = male/1 = female)	Household income (*n* = 1310)
Animal Rights	1.00 ^a^	−0.435 **^,a^	−0.544 **^,a^	−0.291 **^,a^	−0.151 ^a^	0.269 **^,a^	−0.148 **^,a^
Anthropocentric		1.00 ^a^	0.432 **^,a^	0.649 **^,a^	0.061 *^,a^	−0.283 **^,a^	0.120 **^,a^
Animal Protection			1.00 ^a^	0.357 **^,a^	−0.104 ^a^	−0.205 **^,a^	0.164 **^,a^
Lay Utilitarian				1.00 ^a^	−0.033 **^,a^	−0.310 **	0.117 **^,a^

* *p* < 0.05, ** *p* < 0.01. The reduced sample size in the Household income column is because respondents who did not know or did not want to reveal the annual income of their household were excluded from the analysis. ^a^ Spearman’s correlation coefficients.

**Table 4:** Overview of population segments in Denmark, Germany, and Sweden—results of latent profile analysis of pork consumers (class 1 to class 4) and similar overview among non-pork consumers.

Input Variables in the Latent Profile Analysis	Class 1	Class 2	Class 3	Class 4	Neither Eats nor Buys Pork	Vegetarian	Vegan	Total	*p*-Value (eta^2^)
Denmark (*n* = 1612)									
Population share	19.8%	34.8%	29.8%	11.3%	2.3%	1.6%	0.5%	100%	
Animal rights Mean (s.e.)	12.4 (0.69)	50.3 (0.70)	35.1 (0.89)	72.8 (1.29)	44.6 (3.93)	54.5 (6.09)	64.3 (10.44)	40.8 (0.62)	*p* < 0.000 (0.514)
Animal protection Mean (s.e.)	86.8 (0.70)	63.9 (0.62)	75.0 (0.56)	43.6 (1.59)	69.0 (3.08)	65.3 (5.56)	41.7 (12.55)	69.5 (0.49)	*p* < 0.000 (0.411)
Anthropocentric Mean (s.e.)	70.5 (0.95)	50.0 (0.68)	41.7 (0.84)	15.0 (1.11)	49.5 (3.66)	22.8 (3.80)	20.4 (7.06)	47.0 (0.58)	*p* < 0.000 (0.461)
Attitude strength Mean (s.e.)	0.7 (0.05)	0.6 (0.03)	2.9 (0.03)	3.2 (0.06)	n.a. ^a^	n.a. ^a^	n.a. ^a^	1.6 (0.04)	*p* < 0.000 (0.706)
Germany (*n* = 1606)									
Population share	8.1%	44.9%	25.4%	12.2%	4.0%	4.0%	1.4%	100%	
Animal rights Mean (s.e.)	7.5 (0.86)	46.8 (0.74)	46.3 (1.11)	65.3 (1.62)	61.2 (2.71)	67.3 (3.07)	82.3 (5.42)	47.6 (0.63)	*p* < 0.000 (0.305)
Animal protection Mean (s.e.)	81.7 (1.11)	57.1 (0.64)	69.6 (0.83)	26.5 (1.11)	56.1 (3.07)	41.9 (3.48)	44.1 (8.46)	57.7 (0.58)	*p* < 0.000 (0.450)
Anthropocentric Mean (s.e.)	68.0 (1.33)	49.1 (0.63)	32.3 (0.99)	17.3 (1.11)	29.5 (3.19)	21.9 (3.48)	26.4 (4.32)	40.3 (0.58)	*p* < 0.000 (0.385)
Attitude strength Mean (s.e.)	0.6 (0.06)	0.6 (0.02)	2.7 (0.03)	3.0 (0.05)	n.a.^a^	n.a.^a^	n.a.^a^	1.5 (0.03)	*p* < 0.000 (0.735)
Sweden (*n* = 1613)									
Population share	21.0%	24.2%	36.5%	9.5%	3.5%	3.6%	1.7%	100%	
Animal rights Mean (s.e.)	15.8 (0.88)	48.6 (0.85)	42.4 (0.86)	73.0 (1.88)	60.0 (3.28)	65.5 (2.91)	84.7 (4.11)	44.4 (0.65)	*p* < 0.000 (0.446)
Animal protection Mean (s.e.)	85.5 (0.66)	57.4 (0.73)	68.5 (0.55)	26.0 (1.48)	48.3 (3.33)	50.2 (3.39)	31.4 (5.50)	63.3 (0.57)	*p* < 0.000 (0.542)
Anthropocentric Mean (s.e.)	64.8 (0.87)	48.6 (0.72)	32.5 (0.70)	12.6 (1.07)	35.2 (3.04)	27.0 (2.78)	17.0 (3.53)	40.9 (0.57)	*p* < 0.000 (0.491)
Attitude strength Mean (s.e.)	1.4 (0.06)	0.6 (0.03)	3.0 (0.03)	3.2 (0.07)	n.a. ^a^	n.a. ^a^	n.a. ^a^	2.0 (0.04)	*p* < 0.000 (0.622)

s.e., standard error. *p*-values and eta^2^ values are from analysis of variance. If respondents within the subpopulations that do not eat pork, are vegetarians, or vegans purchased pork for others in the family, they were included in one of the four classes (class 1 to 4). Weighted mean values and shares are reported in the table using post-stratification weights as described in the data analysis section. One respondent (a pork consumer) in Germany unfortunately did not receive the attitude strength statements. Therefore, the total sample in this analysis is 1606 (instead of 1607). ^a^ n.a., not applicable in the three sub-populations that neither eat nor purchase pork because they were not given all the attitude strength questions.

**Table 5:** Socio-demographic characteristics of the four pork consumer segments (shares and mean (s.e.) and test of differences).

Socio-Demographic Variables	Class 1 (%)	Class 2 (%)	Class 3 (%)	Class 4 (%)	*p*-Value (Χ^2^(df))
Denmark (*n* = 1536)					
Gender					
Male	0.691	0.512	0.454	0.243	*p* < 0.001 (104.5(3))
Female	0.309	0.488	0.546	0.757	
Income					
Highest quintiles	0.318	0.122	0.223	0.086	*p* < 0.01 (13.6(3))
Other income groups	0.682	0.878	0.777	0.914	
Age (in years) Mean (s.e.)	49.7 (0.91)	49.6 (0.81)	53.4 (0.79)	50.1 (1.39)	*p* < 0.001 (49.6(3))
Germany (*n* = 1474)					
Gender					
Male	0.831	0.493	0.416	0.271	*p* < 0.001 (87.6(3))
Female	0.169	0.507	0.584	0.729	
Household income					
Highest quintiles	0.361	0.188	0.257	0.179	*p* < 0.001 (48.5(3))
Other income groups	0.639	0.812	0.743	0.821	
Age (in years) Mean (s.e.)	55.3 (0.97)	47.5 (0.59)	51.0 (0.73)	50.4 (0.97)	*p* < 0.01 (14.8(3))
Sweden (*n* = 1482)					
Gender					
Male	0.779	0.592	0.359	0.198	*p* < 0.001 (221.8(3))
Female	0.221	0.408	0.641	0.802	
Income					
Highest quintiles	0.323	0.150	0.208	0.150	*p* < 0.001 (20.2(3))
Other income groups	0.677	0.850	0.792	0.850	
Age (in years) Mean (s.e.)	55.783 (0.86)	46.587 (0.94)	54.416 (0.71)	49.628 (1.43)	*p* < 0.001 (65.9(3))

s.e., standard error.

## Data Availability

Data and data codebook are attached as supplementary material (Supplementary materials Files S5 to S8).

## Supplementary Materials

The following are available online at https://www.mdpi.com/2076-2615/11/2/329/s1, File S1: Language-specific versions of the animal ethics questionnaire statements, File S2: Assessment of measurement invariance of the animal ethics orientations, File S3: Latent profile analysis model search results, File S4: Adjusted and unadjusted test of differences between segments in purchase frequency of three types of pork—in Denmark, Germany, and Sweden.

## References

[1] Schmid O., Kilschberger R. (2010). Overview of Animal Welfare Standards and Initiatives in Selected EU and Third Countries. http://ifsa.boku.ac.at/cms/fileadmin/Proceeding2010/2010_WS4.5_Schmid.pdf.

[2] Vogeler C.S. (2019). Why do farm animal welfare regulations vary between EU member states? A comparative analysis of societal and party political determinants in France, Germany, Italy, Spain, and the UK. J. Common Market Stud..

[3] Sandøe P., Christensen T. (2018). Farm Animal Welfare in Europe: From Legislation to Labelling, Working Paper. https://dyreetik.ku.dk/dokumenter/forskningsprojekter/From_legislation_to_labelling.pdf.

[4] European Commission (2016). Attitudes of Europeans towards Animal Welfare. http://ec.europa.eu/COMMFrontOffice/publicopinion/index.cfm/Survey/getSurveyDetail/instruments/SPECIAL/surveyKy/2096.

[5] Christensen T., Denver S., Sandøe P. (2019). How best to improve farm animal welfare? Four main approaches viewed from an economic perspective. Anim. Welf..

[6] Heerwagen L.R., Mørkbak M.R., Denver S., Sandøe P., Christensen T. (2015). The role of quality labels in market-driven animal welfare. J. Agric. Environ. Ethics.

[7] Vogeler C.S. (2019). Market-Based governance in farm animal welfare—A comparative analysis of public and private policies in Germany and France. Animals.

[8] Spain C.V., Freund D., Mohan-Gibbons H., Meadow R.G., Beacham L. (2018). Are they buying it? United States consumers’ changing attitudes toward more humanely raised meat, eggs, and dairy. Animals.

[9] Janssen M., Busch C., Rodiger M., Hamm U. (2016). Motives of consumers following a vegan diet and their attitudes towards animal agriculture. Appetite.

[10] Lund T.B., McKeegan D.E.F., Cribbin C., Sandøe P. (2016). Animal ethics profiling of vegetarians, vegans and meat eaters. Anthrozoos.

[11] Rothgerber H. (2015). Underlying differences between conscientious omnivores and vegetarians in the evaluation of meat and animals. Appetite.

[12] Palmer C., Sandøe P., Appleby M.C., Olsson I.A.S., Galindo F. (2018). Animal Ethics. Animal Welfare.

[13] Garner R. (2005). Animal Ethics.

[14] Lund T.B., Kondrup S.V., Sandøe P. (2019). A multidimensional measure of animal ethics orientation—Developed and applied to a representative sample of the Danish public. PLoS ONE.

[15] Hölker S., Meyer-Höfer M., Spiller A. (2019). Inclusion of animal ethics into the consumer. Value-Attitude system using the example of game meat consumption. Food Ethics.

[16] Frey U.J., Pirscher F. (2018). Willingness to pay and moral stance: The case of farm animal welfare in Germany. PLoS ONE.

[17] Cembalo L., Caracciolo F., Lombardi A., Del Giudice T., Grunert K., Cicia G. (2016). Determinants of individual attitudes toward animal welfare-friendly food products. J. Agric. Environ. Ethics.

[18] Boogaard B.K., Oosting S.J., Bock B.B. (2006). Elements of societal perception of farm animal welfare: A quantitative study in The Netherlands. Livest. Sci..

[19] Miranda-de la Lamaa G.C., Estévez-Moren L.X., Sepúlvedac W.S., Estrada-Chaveroa M.C., Rayas-Amora A.A., Villarroel M., María G.A. (2017). Mexican consumers’ perceptions and attitudes towards farm animal welfare and willingness to pay for welfare friendly meat products. Meat Sci..

[20] Verbeke W., Pérez-Cuetoa F.J., de Barcellos M., Krystallis A., Grunert K. (2010). European citizen and consumer attitudes and preferences regarding beef and pork. Meat Sci..

[21] Maria A. (2016). Public perception of farm animal welfare in Spain. Livest. Sci..

[22] Clark B., Stewart G.B., Panzone L.A., Kyriazakis I., Frewer L.J. (2017). Citizens, consumers and farm animal welfare: A meta-analysis of willingness-to-pay studies. Food Policy.

[23] Mørkbak M., Nordström J. (2009). The impact of information on consumer preferences for different animal food production methods. J. Consum. Policy.

[24] Herzog H., Betchart N.S., Pittman R.B. (1991). Gender, sex role orientation, and attitudes toward animals. Anthrozoos.

[25] Wuensch K.L., Jenkins K.W., Poteat G.M. (2002). Misanthropy, idealism, and attitudes towards animals. Anthrozoos.

[26] Kendall H.A., Lobao L.M., Sharp J.S. (2006). Public concern with animal well-being: Place, social structural location, and individual experience. Rural Sociol..

[27] Howe L., Krosnick J.A. (2017). Attitude strength. Annu. Rev. Psychol..

[28] Bhattacherjee A., Sanford C. (2009). The intention–behaviour gap in technology usage: The moderating role of attitude strength. Behav. Inf. Technol..

[29] Priester J.R., Nayakankuppam D., Fleming M.A., Godek J. (2004). The A2SC2 model: The influence of attitudes and attitude strength on consideration and choice. J. Consum. Res..

[30] Sandøe P., Hansen H.O., Rhode H.L.H., Houe H., Palmer C., Forkman B., Christensen T. (2020). Benchmarking farm animal welfare—A novel tool for cross-country comparison applied to pig production and pork consumption. Animals.

[31] Milfont T.L., Fischer R. (2010). Testing measurement invariance across groups: Applications in cross-cultural research. Int. J. Psychol. Res..

[32] Gadermann A., Guhn M., Zumbo B. (2012). Estimating ordinal reliability for Likert-type and ordinal item response data: A conceptual, empirical, and practical guide. Pract. Assess. Res. Eval..

[33] Berlin K.S., Williams N.A., Parra G.R. (2014). An introduction to latent variable mixture modeling (Part 1: Overview and cross-sectional latent class and latent profile analyses. J. Pediatr. Psychol..

[34] Nylund-Gibson K., Grimm R.P., Masyn K.E. (2019). Prediction from latent classes: A demonstration of different approaches to include distal outcomes in mixture models. Struct. Equ. Modeling Multidiscip. J..

[35] Bauer D.J., Curran P.J. (2003). Over extraction of latent trajectory classes: Much ado about nothing? Reply to Rindskopf (2003), Muthen (2003), and Cudeck and Henly (2003). Psychol. Methods.

[36] Akaike H. (1987). Factor analysis and the AIC. Psychometrika.

[37] Sclove S.L. (1987). Application of model-selection criteria to some problems in multivariate analysis. Psychometrika.

[38] Lo Y., Mendell N., Rubin D.B. (2001). Testing the number of components in a normal mixture. Biometrika.

[39] Ramaswany V., De Sarbo W., Reibstein D., Robinson W. (1993). An empirical pooling approach for estimating marketing mix elasticities with PIMS data. Mark. Sci..

[40] Asparouhov T., Muthén B. (2014). Auxiliary variables in mixture modeling: Using the bch method in mplus to estimate a distal outcome model and an arbitrary secondary model. Mplus Web Notes.

[41] Miranda-de la Lama G.C., Sepúlveda W.S., Villarroel M., María G.A. (2013). Attitudes of meat retailers to animal welfare in Spain. Meat Sci..

[42] Saatkamp H.W., Vissers L.S.M., van Horne P.L.M., de Jong I.C. (2019). Transition from conventional broiler meat to meat from production concepts with higher animal welfare: Experiences from The Netherlands. Animals.

[43] Garner R. (2017). Animals and democratic theory: Beyond an anthropocentric account. Contemp. Political Theory.

[44] Loughnan S., Bastian B., Haslam N. (2014). The psychology of eating animals. Curr. Dir. Psychol. Sci..

[45] Piazza J., Ruby M.B., Loughnan S., Luong M., Kulik J., Watkins H.M., Seigerman M. (2015). Rationalizing meat consumption. The 4Ns. Appetite.

[46] Kunst J.R., Hohle S.M. (2016). Meat eaters by dissociation: How we present, prepare and talk about meat increases willingness to eat meat by reducing empathy and disgust. Appetite.

[47] Kreuter F., Presser S., Tourangeau R. (2008). Social desirability bias in CATI, IVR, and web surveys: The effects of mode and question sensitivity. Public Opin. Q..

[48] Davern M. (2013). Nonresponse rates are a problematic indicator of nonresponse bias in survey research. Health Serv. Res..

[49] Davern M., McAlpine D., Beebe T.J., Ziegenfuss J., Rockwood T., Call K.T. (2010). Are lower response rates hazardous to your health survey? An analysis of three state telephone health surveys. Health Serv. Res..

