# Supplementary material for: Moral Convictions and Meat Consumption—A Comparative Study of the Animal Ethics Orientations of Consumers of Pork in Denmark, Germany, and Sweden
Source: Animals (Basel). 2021 Jan 28;11(2):329. doi: 10.3390/ani11020329 (PMC7912257; doi:10.3390/ani11020329)
Supplement: Supplementary file 1 [file animals-11-00329-s001.zip › supple/Supplementary File 2.docx]

**Supplementary Document 1**

**Assessment of measurement invariance of the animal ethics orientations**

**METHODS**

We evaluated the validity of the measure in the three study countries (Denmark, Germany, and Sweden) by analyses of measurement invariance [1].

Using multi-group confirmatory factor analysis (CFA), we assessed whether the measure exhibited configural invariance, metric invariance, and scalar invariance. These invariance models impose increasing levels of restriction on the data modeling. The scalar invariance model is the most restrictive, implying that the same factorial structure exists and that item factor loadings and intercepts are equivalent in all populations. If scalar invariance is supported, the construct is similar across populations and the scores on the latent constructs can be compared [2]. For each country we also assessed whether it is statistically acceptable to construct variables that measure the four orientations as composite scores (i.e. by summing the raw scores of the factor-specific items) by assessing the fit of the so-called parallel model where factor loadings and error variances of the items are constrained to be equal [2]. We were interested in examining the fit of the parallel model, so provided that it is acceptable, the four orientations could be rescaled to a more easily interpretable score ranging between 0 and 100, where 0 indicates a very low, 100 a very high and 50 a medium propensity to subscribe to the animal ethics orientation in question.

In all CFA models, we used robust maximum likelihood estimation (MLR estimator in MPLUS v. 8.4), where the standard error estimates of maximum likelihood parameters and χ^2^ test statistics are robust to non-normality. Although the 12 input items were ordinal variables, we decided not to use an estimator that takes this into consideration (by calculating polychoric correlations), because we wanted to employ composite scores, as described above, where it is assumed that the values of the items are equidistant. Treating ordinal indicators as continuous is usually acceptable when there are five or more response categories [3]. We did, however, re-run the analysis of measurement invariance with an estimator that relies on polychoric correlations (the WLSMV estimator in MPLUS v. 8.4), and similar results were found. To assess the model fit of the CFA models we reported χ^2^ values and degree of freedom (df), the standardized root mean square residual (SRMR), the comparative fit index (CFI), and the Tucker-Lewis index (TLI). The root mean square error of approximation (RMSEA) was not reported, since recent research shows that the unstandardized (effect size) properties of RMSEA makes it less accurate where the detection of close model fit is concerned compared with SRMR, especially when data are non-normal [4]. We took SRMR values below 0.08 and CFI/TLI values above 0.90 to indicate acceptable fit [5,6].

| **Table S2.** Confirmatory factor analysis of the four-dimensional measure of Animal Ethics Orientation and test of measurement invariance across three countries (Denmark, Germany, and Sweden) |
| --- |
| \| COUNTRY-SPECIFIC FIT STATISTICS OF THE FOUR-FACTOR MODEL \| \| \| \| \| \| \| \| --- \| --- \| --- \| --- \| --- \| --- \| --- \| \|  \| χ^^ \| df \| p-value \| CFI \| TLI \| SRMR \| \| DENMARK (n=1612) \|  \|  \|  \|  \|  \|  \| \| Congeneric model \| 296.2 \| 48 \| <0.000 \| 0.968 \| 0.956 \| 0.043 \| \| Parallel model \| 603.4 \| 64 \| <0.000 \| 0.930 \| 0.928 \| 0.075 \| \| SWEDEN (n=1613) \|  \|  \|  \|  \|  \|  \| \| Congeneric model \| 264.4 \| 48 \| <0.000 \| 0.972 \| 0.962 \| 0.035 \| \| Parallel model \| 472.0 \| 64 \| <0.000 \| 0.947 \| 0.946 \| 0.045 \| \| GERMANY (n=1607) \|  \|  \|  \|  \|  \|  \| \| Congeneric model \| 422.8 \| 48 \| <0.000 \| 0.951 \| 0.933 \| 0.048 \| \| Parallel model \| 795.8 \| 64 \| <0.000 \| 0.905 \| 0.902 \| 0.063 \| |
| \| TEST OF INVARIANCE ACROSS THE THREE COUNTRIES (n=4832) \| \| \| \| \| \| \| \| \| \| \| --- \| --- \| --- \| --- \| --- \| --- \| --- \| --- \| --- \| --- \| \|  \| χ^^ \| df \| p-value \| CFI \| ΔCFI \| TLI \| ΔTLI \| SRMR \| ΔSRMR \| \| Configural \| 981.7 \| 144 \| <0.000 \| 0.964 \|  \| 0.950 \|  \| 0.042 \|  \| \| Metric \| 1116.5 \| 160 \| <0.000 \| 0.959 \| -0.005 \| 0.949 \| -0.001 \| 0.054 \| 0.012 \| \| Scalar \| 1266.7 \| 176 \| <0.000 \| 0.953 \| -0.011 \| 0.947 \| -0.003 \| 0.059 \| 0.015 \| |

**RESULTS**

Results from confirmatory factor analysis laid out in the upper part of **Table 1** show that the so-called congeneric model, where the hypothesized four latent factors are tested, provides an acceptable fit with the data in all three countries, as the CFI/TLI indices are well above the threshold of 0.90 and have SRMR below 0.08. We then went on to look at measurement invariance across the three countries (see the lower part of **Table 1**). There was support for configural invariance, as the fit indices gave acceptable values with CFI/TLI values at.964 and.950 and an SRMR value below 0.08. Relatively modest changes in the fit indices (ΔCFI -0.005; ΔTLI -0.001; ΔSRMR 0.015) were introduced when the factor loadings were constrained to be equal across the countries (metric invariance). Adding the constraint of equal intercepts across the three countries (scalar invariance) only produced minor reductions in the fit indices (ΔCFI -0.011; ΔTLI -0.003; ΔSRMR 0.015). Since the scalar invariance model gave an acceptable fit with the data, it is appropriate to compare the three countries on the four latent scales.

Finally, we examined the fit of the parallel model to see whether it is justifiable to construct variables that measure the four orientations as composite scores. Country-specific CFA results from the parallel model (upper part of **Table 1**) returned acceptable values (CFI/TLI values are over 0.90 and SRMR values are below 0.08), although it should be mentioned that there is a clear decrease in the CFI/TLI values, and increase in the SRMR values, in Denmark and Germany. Still, in order to introduce latent variables that are easier to interpret, we decided to use composite scores (i.e. where the ethics orientation variables were scaled to range from 0 to 100) in the subsequent analyses (reported in the article in Table 1, Table 2, and Table 3). The tests of difference in the prevalence of the orientations across countries and results from latent profile analysis are substantially similar irrespective of whether composite scales or factor scores are used.

References

1. Milfont, T.L., & Fischer, R. (2010). Testing measurement invariance across groups: Applications in cross-cultural research. *International Journal of Psychological Research*, 3, 111-121.
2. Graham, J. (2006). Congeneric and (Essentially) Tau-Equivalent Estimates of Score Reliability. *Educational and Psychological Measurement*, 66, 930–944. [https://doi.org/10.1177/0013164406288165](https://doi.org/10.1177%2F0013164406288165)
3. Rhemtulla, M., Brosseau-Liard, P.E., Savalei, V. (2012). When Can Categorical Variables Be Treated as Continuous? A Comparison of Robust Continuous and Categorical SEM Estimation Methods Under Suboptimal Conditions. *Psychological Methods*, 17, 354–373. https://doi.org/10.1037/a0029315 PMID: 22799625
4. Maydeu-Olivares, A., Shi, D., & Rosseel, Y. (2018). Assessing Fit in Structural Equation Models: A Monte-Carlo Evaluation of RMSEA Versus SRMR Confidence Intervals and Tests of Close Fit. *Structural Equation Modeling. A Multidisciplinary Journal,* 25, 389-402. <https://doi.org/10.1080/10705511.2017.1389611>
5. Hu, L.T., & Bentler, P.M. (1999). Cutoff Criteria for Fit Indexes in Covariance Structure Analysis: Conventional Criteria Versus New Alternatives. *Structural Equation Modeling: A Multidisciplinary Journal*, 6, 1–55. <https://doi.org/10.1080/10705519909540118>
6. Kline, R.B. (2005). *Principles and practice of structural equation modeling*. 2nd ed. New York US: Guilford Press.
