# Supplementary material for: Moral Convictions and Meat Consumption—A Comparative Study of the Animal Ethics Orientations of Consumers of Pork in Denmark, Germany, and Sweden
Source: Animals (Basel). 2021 Jan 28;11(2):329. doi: 10.3390/ani11020329 (PMC7912257; doi:10.3390/ani11020329)
Supplement: Supplementary file 1 [file animals-11-00329-s001.zip › supple/Supplementary File 3.docx]

**Supplementary material 3**

**Results from model search in the three countries.**

| **Table S3.** Latent profile analysis - model search results |
| --- |
| \| Denmark (N=1536) \| \| \| \| \| \| \| --- \| --- \| --- \| --- \| --- \| --- \| \|  \| Best Loglikeli-hood (H0 value ) \| Entropy \| AIC \| Sample size adj. BIC \| LMR- mendel/rubin adj. LRT \| \| 1 class \| -23652.791 \| not applicable \| 47321.582 \| 47338.863 \| not applicable \| \| 2 classes \| -23291.592 \| 0.635 \| 46609.185 \| 46637.267 \| 0.000 \| \| 3 classes \| -23097.923 \| 0.763 \| 46231.845 \| 46270.729 \| 0.000 \| \| 4 classes \| -22973.020 \| 0.721 \| 45992.040 \| 46041.724 \| **0.164** \| \| 5 classes \| -22890.429 \| 0.754 \| 45836.859 \| 45897.344 \| 0.000 \| \| 6 classes \| -22845.913 \| 0.785 \| 45757.826 \| 45829.112 \| 0.0005 \| |
| \| Germany (N=1474) \| \| \| \| \| \| \| --- \| --- \| --- \| --- \| --- \| --- \| \|  \| Best Loglikeli-hood (H0 value ) \| Entropy \| AIC \| Sample size adj. BIC \| LMR- mendel/rubin adj. LRT \| \| 1 class \| -22938.857 \| not applicable \| 45893.714 \| 45910.666 \| not applicable \| \| 2 classes \| -22479.064 \| 0.781 \| 44984.127 \| 45011.675 \| 0.000 \| \| 3 classes \| -22377.239 \| 0.760 \| 44790.477 \| 44828.620 \| 0.000 \| \| 4 classes \| -22307.118 \| 0.720 \| 44660.236 \| 44708.974 \| 0.003 \| \| 5 classes \| -22272.839 \| 0.751 \| 44601.678 \| 44661.011 \| **0.223** \| \| 6 classes \| -22232.414 \| 0.777 \| 44530.828 \| 44600.756 \| **0.154** \| |
| \| Sweden (N=1482) \| \| \| \| \| \| \| --- \| --- \| --- \| --- \| --- \| --- \| \|  \| Best Loglikeli-hood (H0 value) \| Entropy \| AIC \| Sample size adj. BIC \| LMR- mendel/rubin adj. LRT \| \| 1 class \| -23077.672 \| not applicable \| 46171.344 \| 46188.340 \| not applicable \| \| 2 classes \| -22688.912 \| 0.694 \| 45403.824 \| 45431.442 \| 0.000 \| \| 3 classes \| -22545.031 \| 0.716 \| 45126.061 \| 45164.301 \| 0.006 \| \| 4 classes \| -22417.330 \| 0.685 \| 44880.659 \| 44929.521 \| 0.000 \| \| 5 classes \| -22343.614 \| 0.695 \| 44743.227 \| 44802.712 \| 0.0021 \| \| 6 classes \| -22289.293 \| 0.745 \| 44644.586 \| 44714.692 \| 0.06 \| |
