# Supplementary material for: Moral Convictions and Meat Consumption—A Comparative Study of the Animal Ethics Orientations of Consumers of Pork in Denmark, Germany, and Sweden
Source: Animals (Basel). 2021 Jan 28;11(2):329. doi: 10.3390/ani11020329 (PMC7912257; doi:10.3390/ani11020329)
Supplement: Supplementary file 1 [file animals-11-00329-s001.zip › supple/Supplementary File 4.docx]

**Supplementary material 4**

**Test of differences between segments in purchase frequency of pork**

| **Table S4.** Adjusted and unadjusted test of differences between segments in purchase frequency of three types of pork – in Denmark, Germany, and Sweden | | | |
| --- | --- | --- | --- |
|  | Denmark  X^2^(df) | Germany  X^2^(df) | Sweden  X^2^(df) |
| **Welfare pork type 1** |  |  |  |
| Unadjusted test statistics^A^ | 29.13(3)*** | 92.30(3)*** | Not applicable |
| Adjusted test statistics^B^ | 13.14(3)** | 40.37(3)*** | Not applicable |
| **Welfare pork type 2** |  |  |  |
| Unadjusted test statistics^A^ | 52.44(3)*** | 37.857(3)*** | 39.10(3)*** |
| Adjusted test statistics^B^ | 52.66(3)*** | 24.75(3)*** | 8.93(3)* |
| **Conventional pork** |  |  |  |
| Unadjusted test statistics^A^ | 75.46(3)*** | 75.46(3)*** | 21.5(3)*** |
| Adjusted test statistics^B^ | 46.78(3)*** | 23.00(3)*** | 9.35(3)* |
| ^A^ Test of difference between segments (classes 1 to 4) using the BCH method in Mplus (Asparouhov, T., & Muthén, 2018). Wald’s chi-square values (degree of freedom) are reported.  ^B^ Test of difference between segments (classes 1 to 4) using the BCH method in Mplus (Asparouhov, T., & Muthén, 2018) after controlling for the effect on pork purchase from three sociodemographic variables (age, gender, household income), and 11 variables prompting for other important factors consumers consider when purchasing pork (easy to purchase, appropriate pieces/cuts, domestically produced, taste, inexpensive, low fat, low environmental burden, low climate burden, food safety, no use of antibiotics, no use of genetically modified feedstuff). Wald’s chi-square values (degree of freedom) are reported. In Germany the climate burden variable was not inserted as predictor in adjusted analysis, as standard errors could not be computed with this variable in the model (because of very low prevalence<5%).  * p<0.05  ** p<0.01  *** p<0.001 | | | |
|  | | | |
