# Supplementary material for: Moral Convictions and Meat Consumption—A Comparative Study of the Animal Ethics Orientations of Consumers of Pork in Denmark, Germany, and Sweden
Source: Animals (Basel). 2021 Jan 28;11(2):329. doi: 10.3390/ani11020329 (PMC7912257; doi:10.3390/ani11020329)
Supplement: Supplementary file 1 [file animals-11-00329-s001.zip › supple/Supplementary File 8 (Country specific codebook).docx]

|  | |  |  | |  |  | |
| --- | --- | --- | --- | --- | --- | --- | --- |
|  | q1 |  |  | q1 |  |  | q1 |
| Position | 2 |  | Position | 3 |  | Position | 3 |
| Label | Svinekød kan spises i mange forskellige former, fx i hele kødudskæringer, som hakket kød, bacon eller som ingrediens i pålæg, pølser og frikadeller. Spiser du én eller flere former for svinekød? |  | Label | Schweinefleisch kann in vielen verschiedenen Formen gegessen werden, z. B. als ganze Teilstücke, als Hackfleisch, als Bacon oder als Zutat in Aufschnitten, Würsten oder Bouletten. Essen Sie eine oder mehrere Arten von Schweinefleisch? |  | Label | Fläskkött kan äta i många olika former, t.ex. i hela köttstycken, som malet kött, bacon, eller som ingrediens i pålägg, korv och köttbullar. Köper du/ditt hushåll en eller flera sorters fläskkött? |
| Type | Numeric |  | Type | Numeric |  | Type | Numeric |
| Format | F1 |  | Format | F1 |  | Format | F1 |
| Measurement | Nominal |  | Measurement | Nominal |  | Measurement | Nominal |
| Role | Input |  | Role | Input |  | Role | Input |
| 1 | Ja |  | 1 | Ja |  | 1 | Ja |
| 2 | Nej, jeg spiser aldrig svinekød |  | 2 | Nein, ich esse niemals Schweinefleisch. |  | 2 | Nej, jag äter aldrig fläskkött |
| 3 | Nej, jeg spiser hovedsageligt en vegetarisk kost (dvs. ingen kød - kun mælkeprodukter, æg og ost) |  | 3 | Nein, ich ernähre mich hauptsächlich vegetarisch (d. h. kein Fleisch - ausschließlich Milchprodukte, Eier und Käse). |  | 3 | Nej, jag äter huvudsakligen vegetariskt (dvs. inget kött – endast mjölkprodukter, ägg och ost) |
| 4 | Nej, jeg spiser hovedsageligt en vegansk kost (dvs. hverken kød, mælkeprodukter, æg eller ost) |  | 4 | Nein, ich ernähre mich hauptsächlich vegan (d. h. weder Fleisch noch Milchprodukte, Eier oder Käse). |  | 4 | Nej, jag äter huvudsakligen veganskt (dvs. varken kött, mjölkprodukter, ägg eller ost) |
|  |  |  |  |  |  |  |  |
|  | |  |  | |  |  | |
|  | q2 |  |  | q2 |  |  | q2 |
| Position | 3 |  | Position | 4 |  | Position | 4 |
| Label | Køber du nogensinde svinekød til andre personer i hustanden (f.eks. din samlever/ægtefælle eller børn) eller gæster? |  | Label | Kaufen Sie jemals Schweinefleisch für andere Personen im Haushalt (z. B. Ihren Lebenspartner / Ehepartner oder Kinder) oder für Gäste? |  | Label | Köper du någonsin fläskkött till andra personer i ditt hushåll (t.ex. din partner / make/maka eller barn) eller gäster? |
| Type | Numeric |  | Type | Numeric |  | Type | Numeric |
| Format | F1 |  | Format | F1 |  | Format | F1 |
| Measurement | Nominal |  | Measurement | Nominal |  | Measurement | Nominal |
| Role | Input |  | Role | Input |  | Role | Input |
| 1 | Ja, det gør jeg ofte |  | 1 | Ja, oft |  | 1 | Ja, det gör jag ofta |
| 2 | Ja, det gør jeg en gang imellem |  | 2 | Ja, ab und zu |  | 2 | Ja, det gör jag ibland |
| 3 | Nej, det gør jeg aldrig eller ret sjældent |  | 3 | Nein, nie oder eher selten |  | 3 | Nej, det gör jag aldrig eller ganska sällan |
| System |  |  | System |  |  | System |  |
|  |  |  |  |  |  |  |  |
|  | |  |  | |  |  | |
|  | q3 |  |  | q3 |  |  | q3 |
| Position | 4 |  | Position | 5 |  | Position | 5 |
| Label | Det burde forbydes ved lov, at mennesker bruger dyr. - Her følger der en række udsagn, som udtrykker holdninger til brug af dyr. Du bedes tage stilling til, hvor enig du er i udsagnene. Du kan svare på en skala der går fra ”helt uenig” til ”helt enig”. Nå |  | Label | Die Nutzung von Tieren sollte gesetzlich verboten werden.. - Auf den nächsten beiden Seiten finden Sie eine Reihe von Aussagen zur Nutzung von Tieren. Bitte teilen Sie uns mit, inwiefern Sie diese Auffassungen teilen |  | Label | Det bör vara förbjudet enligt lag för människor att använda djur.. - När djur och djuranvändning nämns i frågorna, tänk på djur som används av människor, t.ex. i samband med jordbruksproduktion, djurförsök eller för visning i cirkusar och djurparker. |
| Type | Numeric |  | Type | Numeric |  | Type | Numeric |
| Format | F1 |  | Format | F1 |  | Format | F1 |
| Measurement | Nominal |  | Measurement | Nominal |  | Measurement | Nominal |
| Role | Input |  | Role | Input |  | Role | Input |
| 1 | Helt uenig |  | 1 | Ich stimme mit der Aussage ganz und gar nicht überein |  | 1 | Instämmer inte alls |
| 2 | Uenig |  | 2 | Lehne ich ab |  | 2 | Instämmer inte |
| 3 | Hverken uenig eller enig |  | 3 | Lehne ich weder ab noch stimme ich zu |  | 3 | Varken instämmer eller instämmer inte |
| 4 | Enig |  | 4 | Stimme zu |  | 4 | Instämmer |
| 5 | Helt enig |  | 5 | Ich stimme mit der Aussage ganz und gar überein |  | 5 | Instämmer helt |
|  |  |  |  |  |  |  |  |
|  | |  |  | |  |  | |
|  | q4 |  |  | q4 |  |  | q4 |
| Position | 5 |  | Position | 6 |  | Position | 6 |
| Label | Det er som udgangspunkt uacceptabelt at mennesker bruger dyr, fordi dyr kan føle smerte, glæde og lignende. - Her følger der en række udsagn, som udtrykker holdninger til brug af dyr. Du bedes tage stilling til, hvor enig du er i udsagnene. Du kan svare p |  | Label | Grundsätzlich ist es inakzeptabel, dass der Mensch Tiere nutzt, da diese Schmerzen, Freude usw. empfinden.. - Auf den nächsten beiden Seiten finden Sie eine Reihe von Aussagen zur Nutzung von Tieren. Bitte teilen Sie uns mit, inwiefern Sie diese Auffassun |  | Label | Det är generellt sett oacceptabelt att människor använder djur eftersom djur kan känna smärta, glädje och liknande... - När djur och djuranvändning nämns i frågorna, tänk på djur som används av människor, t.ex. i samband med jordbruksproduktion, djurförsö |
| Type | Numeric |  | Type | Numeric |  | Type | Numeric |
| Format | F1 |  | Format | F1 |  | Format | F1 |
| Measurement | Nominal |  | Measurement | Nominal |  | Measurement | Nominal |
| Role | Input |  | Role | Input |  | Role | Input |
| 1 | Helt uenig |  | 1 | Ich stimme mit der Aussage ganz und gar nicht überein |  | 1 | Instämmer inte alls |
| 2 | Uenig |  | 2 | Lehne ich ab |  | 2 | Instämmer inte |
| 3 | Hverken uenig eller enig |  | 3 | Lehne ich weder ab noch stimme ich zu |  | 3 | Varken instämmer eller instämmer inte |
| 4 | Enig |  | 4 | Stimme zu |  | 4 | Instämmer |
| 5 | Helt enig |  | 5 | Ich stimme mit der Aussage ganz und gar überein |  | 5 | Instämmer helt |
|  |  |  |  |  |  |  |  |
|  | |  |  | |  |  | |
|  | q5 |  |  | q5 |  |  | q5 |
| Position | 6 |  | Position | 7 |  | Position | 7 |
| Label | Det er som udgangspunkt uacceptabelt at mennesker bruger dyr, fordi dyr er væsner, som sanser og tænker. - Her følger der en række udsagn, som udtrykker holdninger til brug af dyr. Du bedes tage stilling til, hvor enig du er i udsagnene. Du kan svare på e |  | Label | Grundsätzlich ist es inakzeptabel, dass der Mensch Tiere nutzt, da Tiere Wesen sind, die fühlen und denken. - Auf den nächsten beiden Seiten finden Sie eine Reihe von Aussagen zur Nutzung von Tieren. Bitte teilen Sie uns mit, inwiefern Sie diese Auffassun |  | Label | Det är generellt sett oacceptabelt att människor använder djur eftersom djur är varelser som känner och tänker... - När djur och djuranvändning nämns i frågorna, tänk på djur som används av människor, t.ex. i samband med jordbruksproduktion, djurförsök el |
| Type | Numeric |  | Type | Numeric |  | Type | Numeric |
| Format | F1 |  | Format | F1 |  | Format | F1 |
| Measurement | Nominal |  | Measurement | Nominal |  | Measurement | Nominal |
| Role | Input |  | Role | Input |  | Role | Input |
| 1 | Helt uenig |  | 1 | Ich stimme mit der Aussage ganz und gar nicht überein |  | 1 | Instämmer inte alls |
| 2 | Uenig |  | 2 | Lehne ich ab |  | 2 | Instämmer inte |
| 3 | Hverken uenig eller enig |  | 3 | Lehne ich weder ab noch stimme ich zu |  | 3 | Varken instämmer eller instämmer inte |
| 4 | Enig |  | 4 | Stimme zu |  | 4 | Instämmer |
| 5 | Helt enig |  | 5 | Ich stimme mit der Aussage ganz und gar überein |  | 5 | Instämmer helt |
|  |  |  |  |  |  |  |  |
|  | |  |  | |  |  | |
|  | q6 |  |  | q6 |  |  | q6 |
| Position | 7 |  | Position | 8 |  | Position | 8 |
| Label | Det er acceptabelt at bruge dyr til vigtige menneskelige formål (f.eks. medicinsk forskning), hvis det sikres at dyrene ikke oplever unødig stress. - Her følger der en række udsagn, som udtrykker holdninger til brug af dyr. Du bedes tage stilling til, hvo |  | Label | Die Nutzung von Tieren für wichtige menschliche Zielstellungen (wie z. B. der medizinischen Forschung) ist akzeptabel, wenn Tiere vor unnötigem Stress geschützt werden. - Auf den nächsten beiden Seiten finden Sie eine Reihe von Aussagen zur Nutzung von Ti |  | Label | Det är acceptabelt att använda djur för viktiga mänskliga ändamål (t.ex. medicinsk forskning) om det säkerställs att djuren inte upplever otillbörlig stress. - När djur och djuranvändning nämns i frågorna, tänk på djur som används av människor, t.ex. i sa |
| Type | Numeric |  | Type | Numeric |  | Type | Numeric |
| Format | F1 |  | Format | F1 |  | Format | F1 |
| Measurement | Nominal |  | Measurement | Nominal |  | Measurement | Nominal |
| Role | Input |  | Role | Input |  | Role | Input |
| 1 | Helt uenig |  | 1 | Ich stimme mit der Aussage ganz und gar nicht überein |  | 1 | Instämmer inte alls |
| 2 | Uenig |  | 2 | Lehne ich ab |  | 2 | Instämmer inte |
| 3 | Hverken uenig eller enig |  | 3 | Lehne ich weder ab noch stimme ich zu |  | 3 | Varken instämmer eller instämmer inte |
| 4 | Enig |  | 4 | Stimme zu |  | 4 | Instämmer |
| 5 | Helt enig |  | 5 | Ich stimme mit der Aussage ganz und gar überein |  | 5 | Instämmer helt |
|  |  |  |  |  |  |  |  |
|  | |  |  | |  |  | |
|  | q7 |  |  | q7 |  |  | q7 |
| Position | 8 |  | Position | 9 |  | Position | 9 |
| Label | Det er acceptabelt at bruge dyr til vigtige menneskelige formål, hvis det sikres at dyrene ikke oplever unødig smerte og anden lidelse. - Her følger der en række udsagn, som udtrykker holdninger til brug af dyr. Du bedes tage stilling til, hvor enig du er |  | Label | Die Nutzung von Tieren für wichtige menschliche Zielstellungen (wie z. B. der medizinischen Forschung) ist akzeptabel, wenn Tiere vor unnötigen Schmerzen und Leid geschützt werden.. - Auf den nächsten beiden Seiten finden Sie eine Reihe von Aussagen zur N |  | Label | Det är acceptabelt att använda djur för viktiga mänskliga ändamål om det säkerställs att djuren inte upplever onödig smärta och andra sjukdomar.. - När djur och djuranvändning nämns i frågorna, tänk på djur som används av människor, t.ex. i samband med jo |
| Type | Numeric |  | Type | Numeric |  | Type | Numeric |
| Format | F1 |  | Format | F1 |  | Format | F1 |
| Measurement | Nominal |  | Measurement | Nominal |  | Measurement | Nominal |
| Role | Input |  | Role | Input |  | Role | Input |
| 1 | Helt uenig |  | 1 | Ich stimme mit der Aussage ganz und gar nicht überein |  | 1 | Instämmer inte alls |
| 2 | Uenig |  | 2 | Lehne ich ab |  | 2 | Instämmer inte |
| 3 | Hverken uenig eller enig |  | 3 | Lehne ich weder ab noch stimme ich zu |  | 3 | Varken instämmer eller instämmer inte |
| 4 | Enig |  | 4 | Stimme zu |  | 4 | Instämmer |
| 5 | Helt enig |  | 5 | Ich stimme mit der Aussage ganz und gar überein |  | 5 | Instämmer helt |
|  |  |  |  |  |  |  |  |
|  | |  |  | |  |  | |
|  | q8 |  |  | q8 |  |  | q8 |
| Position | 9 |  | Position | 10 |  | Position | 10 |
| Label | Det er acceptabelt at bruge dyr til vigtige menneskelige formål, hvis dyrene har et ordentligt liv. - Her følger der en række udsagn, som udtrykker holdninger til brug af dyr. Du bedes tage stilling til, hvor enig du er i udsagnene. Du kan svare på en ska |  | Label | Die Nutzung von Tieren für wichtige menschliche Zielstellungen ist akzeptabel, wenn die Tiere unter ordentlichen Verhältnissen gehalten werden. - Auf den nächsten beiden Seiten finden Sie eine Reihe von Aussagen zur Nutzung von Tieren. Bitte teilen Sie un |  | Label | Det är acceptabelt att använda djur för viktiga mänskliga ändamål om djuren har ett ordentligt liv. - När djur och djuranvändning nämns i frågorna, tänk på djur som används av människor, t.ex. i samband med jordbruksproduktion, djurförsök eller för visnin |
| Type | Numeric |  | Type | Numeric |  | Type | Numeric |
| Format | F1 |  | Format | F1 |  | Format | F1 |
| Measurement | Nominal |  | Measurement | Nominal |  | Measurement | Nominal |
| Role | Input |  | Role | Input |  | Role | Input |
| 1 | Helt uenig |  | 1 | Ich stimme mit der Aussage ganz und gar nicht überein |  | 1 | Instämmer inte alls |
| 2 | Uenig |  | 2 | Lehne ich ab |  | 2 | Instämmer inte |
| 3 | Hverken uenig eller enig |  | 3 | Lehne ich weder ab noch stimme ich zu |  | 3 | Varken instämmer eller instämmer inte |
| 4 | Enig |  | 4 | Stimme zu |  | 4 | Instämmer |
| 5 | Helt enig |  | 5 | Ich stimme mit der Aussage ganz und gar überein |  | 5 | Instämmer helt |
|  |  |  |  |  |  |  |  |
|  | |  |  | |  |  | |
|  | q9 |  |  | q9 |  |  | q9 |
| Position | 10 |  | Position | 11 |  | Position | 11 |
| Label | Det kan godt forsvares at dyr oplever stress og velfærdsforringelser, hvis det tjener et formål, som er vigtigt nok. - ....Du bedes fortsat svare på en skala fra “Helt uenig” til “Helt enig” |  | Label | Es ist gerechtfertigt, wenn Tiere Stress erleiden oder das Tierwohl eingeschränkt wird, wenn dies einem wichtigen Zweck dient. - ....Bitte geben Sie erneut an, wie sehr Sie der Aussage zustimmen. |  | Label | Att djur upplever stress och försämrad välfärd kan försvaras om det tjänar ett syfte som är tillräckligt viktigt.. - ...….. Vänligen fortsätt att svara på en skala från " Instämmer inte alls" till "Instämmer helt" |
| Type | Numeric |  | Type | Numeric |  | Type | Numeric |
| Format | F1 |  | Format | F1 |  | Format | F1 |
| Measurement | Nominal |  | Measurement | Nominal |  | Measurement | Nominal |
| Role | Input |  | Role | Input |  | Role | Input |
| 1 | Helt uenig |  | 1 | Ich stimme mit der Aussage ganz und gar nicht überein |  | 1 | Instämmer inte alls |
| 2 | Uenig |  | 2 | Lehne ich ab |  | 2 | Instämmer inte |
| 3 | Hverken uenig eller enig |  | 3 | Lehne ich weder ab noch stimme ich zu |  | 3 | Varken instämmer eller instämmer inte |
| 4 | Enig |  | 4 | Stimme zu |  | 4 | Instämmer |
| 5 | Helt enig |  | 5 | Ich stimme mit der Aussage ganz und gar überein |  | 5 | Instämmer helt |
|  |  |  |  |  |  |  |  |
|  | |  |  | |  |  | |
|  | q10 |  |  | q10 |  |  | q10 |
| Position | 11 |  | Position | 12 |  | Position | 12 |
| Label | Det kan godt forsvares at påføre dyr væsentlig smerte, hvis formålet er vigtigt nok (f.eks. medicinsk forskning). - ....Du bedes fortsat svare på en skala fra “Helt uenig” til “Helt enig” |  | Label | Es ist gerechtfertigt, Tieren Schmerz zuzuführen, wenn dies einem wichtigen Zweck (z. B. medizinische Forschung) dient.. - ....Bitte geben Sie erneut an, wie sehr Sie der Aussage zustimmen. |  | Label | Att utsätta djur för betydande smärta kan försvaras om det tjänar ett syfte som är tillräckligt viktigt. - .….. Vänligen fortsätt att svara på en skala från " Instämmer inte alls" till "Instämmer helt" |
| Type | Numeric |  | Type | Numeric |  | Type | Numeric |
| Format | F1 |  | Format | F1 |  | Format | F1 |
| Measurement | Nominal |  | Measurement | Nominal |  | Measurement | Nominal |
| Role | Input |  | Role | Input |  | Role | Input |
| 1 | Helt uenig |  | 1 | Ich stimme mit der Aussage ganz und gar nicht überein |  | 1 | Instämmer inte alls |
| 2 | Uenig |  | 2 | Lehne ich ab |  | 2 | Instämmer inte |
| 3 | Hverken uenig eller enig |  | 3 | Lehne ich weder ab noch stimme ich zu |  | 3 | Varken instämmer eller instämmer inte |
| 4 | Enig |  | 4 | Stimme zu |  | 4 | Instämmer |
| 5 | Helt enig |  | 5 | Ich stimme mit der Aussage ganz und gar überein |  | 5 | Instämmer helt |
|  |  |  |  |  |  |  |  |
|  | |  |  | |  |  | |
|  | q11 |  |  | q11 |  |  | q11 |
| Position | 12 |  | Position | 13 |  | Position | 13 |
| Label | Det kan være acceptabelt at påføre dyr alvorlig smerte, hvis det er nødvendigt for at opnå et vitalt menneskeligt formål (f.eks. medicinsk forskning). - ....Du bedes fortsat svare på en skala fra “Helt uenig” til “Helt enig” |  | Label | Es kann gerechtfertigt sein, Tieren erheblichen Schmerz zuzuführen, wenn dies einem vitalen menschlichen Bedürfnis (z. B. medizinische Forschung) dient. - ....Bitte geben Sie erneut an, wie sehr Sie der Aussage zustimmen. |  | Label | Det kan vara acceptabelt att orsaka allvarlig smärta på djur, om det är nödvändigt för att uppnå ett viktigt mänskligt syfte (t.ex. medicinsk forskning).. - ...….. Vänligen fortsätt att svara på en skala från " Instämmer inte alls" till "Instämmer helt" |
| Type | Numeric |  | Type | Numeric |  | Type | Numeric |
| Format | F1 |  | Format | F1 |  | Format | F1 |
| Measurement | Nominal |  | Measurement | Nominal |  | Measurement | Nominal |
| Role | Input |  | Role | Input |  | Role | Input |
| 1 | Helt uenig |  | 1 | Ich stimme mit der Aussage ganz und gar nicht überein |  | 1 | Instämmer inte alls |
| 2 | Uenig |  | 2 | Lehne ich ab |  | 2 | Instämmer inte |
| 3 | Hverken uenig eller enig |  | 3 | Lehne ich weder ab noch stimme ich zu |  | 3 | Varken instämmer eller instämmer inte |
| 4 | Enig |  | 4 | Stimme zu |  | 4 | Instämmer |
| 5 | Helt enig |  | 5 | Ich stimme mit der Aussage ganz und gar überein |  | 5 | Instämmer helt |
|  |  |  |  |  |  |  |  |
|  | |  |  | |  |  | |
|  | q12 |  |  | q12 |  |  | q12 |
| Position | 13 |  | Position | 14 |  | Position | 14 |
| Label | Vi har ret til at bruge dyr, fordi mennesker er intellektuelt overlegne i forhold til dyrene. - ....Du bedes fortsat svare på en skala fra “Helt uenig” til “Helt enig” |  | Label | Wir haben das Recht, Tiere zu unseren Zwecken zu nutzen, da wir intellektuell überlegen sind. - ....Bitte geben Sie erneut an, wie sehr Sie der Aussage zustimmen. |  | Label | Vi har rätt att använda djur eftersom människor är intellektuellt överlägsna djuren.. - ...….. ….. Vänligen fortsätt att svara på en skala från " Instämmer inte alls" till "Instämmer helt" |
| Type | Numeric |  | Type | Numeric |  | Type | Numeric |
| Format | F1 |  | Format | F1 |  | Format | F1 |
| Measurement | Nominal |  | Measurement | Nominal |  | Measurement | Nominal |
| Role | Input |  | Role | Input |  | Role | Input |
| 1 | Helt uenig |  | 1 | Ich stimme mit der Aussage ganz und gar nicht überein |  | 1 | Instämmer inte alls |
| 2 | Uenig |  | 2 | Lehne ich ab |  | 2 | Instämmer inte |
| 3 | Hverken uenig eller enig |  | 3 | Lehne ich weder ab noch stimme ich zu |  | 3 | Varken instämmer eller instämmer inte |
| 4 | Enig |  | 4 | Stimme zu |  | 4 | Instämmer |
| 5 | Helt enig |  | 5 | Ich stimme mit der Aussage ganz und gar überein |  | 5 | Instämmer helt |
|  |  |  |  |  |  |  |  |
|  | |  |  | |  |  | |
|  | q13 |  |  | q13 |  |  | q13 |
| Position | 14 |  | Position | 15 |  | Position | 15 |
| Label | Menneskehedens interesser er vigtigere end dyrs interesser. - ....Du bedes fortsat svare på en skala fra “Helt uenig” til “Helt enig” |  | Label | Die Interessen des Menschen sind wichtiger als die Interessen der Tiere. - ....Bitte geben Sie erneut an, wie sehr Sie der Aussage zustimmen. |  | Label | Mänsklighetens intressen är viktigare än djurens intressen.- ...….. Vänligen fortsätt att svara på en skala från " Instämmer inte alls" till "Instämmer helt" |
| Type | Numeric |  | Type | Numeric |  | Type | Numeric |
| Format | F1 |  | Format | F1 |  | Format | F1 |
| Measurement | Nominal |  | Measurement | Nominal |  | Measurement | Nominal |
| Role | Input |  | Role | Input |  | Role | Input |
| 1 | Helt uenig |  | 1 | Ich stimme mit der Aussage ganz und gar nicht überein |  | 1 | Instämmer inte alls |
| 2 | Uenig |  | 2 | Lehne ich ab |  | 2 | Instämmer inte |
| 3 | Hverken uenig eller enig |  | 3 | Lehne ich weder ab noch stimme ich zu |  | 3 | Varken instämmer eller instämmer inte |
| 4 | Enig |  | 4 | Stimme zu |  | 4 | Instämmer |
| 5 | Helt enig |  | 5 | Ich stimme mit der Aussage ganz und gar überein |  | 5 | Instämmer helt |
|  |  |  |  |  |  |  |  |
|  | |  |  | |  |  | |
|  | q14 |  |  | q14 |  |  | q14 |
| Position | 15 |  | Position | 16 |  | Position | 16 |
| Label | Vi skal tage hensyn til mennesker frem for dyr. - ....Du bedes fortsat svare på en skala fra “Helt uenig” til “Helt enig” |  | Label | Wir sollten mehr Rücksicht auf Menschen als auf Tiere nehmen.. - ....Bitte geben Sie erneut an, wie sehr Sie der Aussage zustimmen. |  | Label | Vi måste ta hänsyn till människor snarare än djur. - ...….. Vänligen fortsätt att svara på en skala från " Instämmer inte alls" till "Instämmer helt" |
| Type | Numeric |  | Type | Numeric |  | Type | Numeric |
| Format | F1 |  | Format | F1 |  | Format | F1 |
| Measurement | Nominal |  | Measurement | Nominal |  | Measurement | Nominal |
| Role | Input |  | Role | Input |  | Role | Input |
| 1 | Helt uenig |  | 1 | Ich stimme mit der Aussage ganz und gar nicht überein |  | 1 | Instämmer inte alls |
| 2 | Uenig |  | 2 | Lehne ich ab |  | 2 | Instämmer inte |
| 3 | Hverken uenig eller enig |  | 3 | Lehne ich weder ab noch stimme ich zu |  | 3 | Varken instämmer eller instämmer inte |
| 4 | Enig |  | 4 | Stimme zu |  | 4 | Instämmer |
| 5 | Helt enig |  | 5 | Ich stimme mit der Aussage ganz und gar überein |  | 5 | Instämmer helt |
|  |  |  |  |  |  |  |  |
|  | |  |  | |  |  | |
|  | q15 |  |  | q15 |  |  | q15 |
| Position | 16 |  | Position | 17 |  | Position | 17 |
| Label | Animal rights |  | Label | Animal rights |  | Label | <none> |
| Type | Numeric |  | Type | Numeric |  | Type | Numeric |
| Format | F8.2 |  | Format | F8.2 |  | Format | F8.2 |
| Measurement | Scale |  | Measurement | Scale |  | Measurement | Scale |
| Role | Input |  | Role | Input |  | Role | Input |
| Valid | 1612 |  | Valid | 1607 |  | Valid | 1613 |
| Missing | 0 |  | Missing | 0 |  | Missing | 0 |
| Mean | 40,7938 |  | Mean | 47,6125 |  | Mean | 41,6770 |
| Standard Deviation | 25,05810 |  | Standard Deviation | 25,25456 |  | Standard Deviation | 26,36375 |
| Percentile 25 | 25,0000 |  | Percentile 25 | 33,3333 |  | Percentile 25 | 25,0000 |
| Percentile 50 | 41,6667 |  | Percentile 50 | 50,0000 |  | Percentile 50 | 41,6667 |
| Percentile 75 | 58,3333 |  | Percentile 75 | 66,6667 |  | Percentile 75 | 58,3333 |
|  |  |  |  |  |  |  |  |
|  | |  |  | |  |  | |
|  | q16 |  |  | q16 |  |  | q16 |
| Position | 17 |  | Position | 18 |  | Position | 18 |
| Label | Anthropocentric |  | Label | Anthropocentric |  | Label | <none> |
| Type | Numeric |  | Type | Numeric |  | Type | Numeric |
| Format | F8.2 |  | Format | F8.2 |  | Format | F8.2 |
| Measurement | Scale |  | Measurement | Scale |  | Measurement | Scale |
| Role | Input |  | Role | Input |  | Role | Input |
| Valid | 1612 |  | Valid | 1607 |  | Valid | 1613 |
| Missing | 0 |  | Missing | 0 |  | Missing | 0 |
| Mean | 47,0371 |  | Mean | 40,3328 |  | Mean | 41,4652 |
| Standard Deviation | 23,25527 |  | Standard Deviation | 23,28887 |  | Standard Deviation | 23,06218 |
| Percentile 25 | 33,3333 |  | Percentile 25 | 25,0000 |  | Percentile 25 | 25,0000 |
| Percentile 50 | 50,0000 |  | Percentile 50 | 41,6667 |  | Percentile 50 | 41,6667 |
| Percentile 75 | 58,3333 |  | Percentile 75 | 58,3333 |  | Percentile 75 | 58,3333 |
|  |  |  |  |  |  |  |  |
|  | |  |  | |  |  | |
|  | q17 |  |  | q17 |  |  | q17 |
| Position | 18 |  | Position | 19 |  | Position | 19 |
| Label | Animal protection |  | Label | Animal protection |  | Label | <none> |
| Type | Numeric |  | Type | Numeric |  | Type | Numeric |
| Format | F8.2 |  | Format | F8.2 |  | Format | F8.2 |
| Measurement | Scale |  | Measurement | Scale |  | Measurement | Scale |
| Role | Input |  | Role | Input |  | Role | Input |
| Valid | 1612 |  | Valid | 1607 |  | Valid | 1613 |
| Missing | 0 |  | Missing | 0 |  | Missing | 0 |
| Mean | 69,4826 |  | Mean | 57,7186 |  | Mean | 64,5846 |
| Standard Deviation | 19,67312 |  | Standard Deviation | 23,12933 |  | Standard Deviation | 22,68195 |
| Percentile 25 | 58,3333 |  | Percentile 25 | 50,0000 |  | Percentile 25 | 50,0000 |
| Percentile 50 | 75,0000 |  | Percentile 50 | 58,3333 |  | Percentile 50 | 66,6667 |
| Percentile 75 | 75,0000 |  | Percentile 75 | 75,0000 |  | Percentile 75 | 75,0000 |
|  |  |  |  |  |  |  |  |
|  | |  |  | |  |  | |
|  | q17a |  |  | q17a |  |  | q17a |
| Position | 18 |  | Position | 19 |  | Position | 19 |
| Label | Lay utilitarian |  | Label | Lay utilitarian |  | Label | <none> |
| Type | Numeric |  | Type | Numeric |  | Type | Numeric |
| Format | F8.2 |  | Format | F8.2 |  | Format | F8.2 |
| Measurement | Scale |  | Measurement | Scale |  | Measurement | Scale |
| Role | Input |  | Role | Input |  | Role | Input |
| Valid | 1612 |  | Valid | 1607 |  | Valid | 1613 |
| Missing | 0 |  | Missing | 0 |  | Missing | 0 |
| Mean | 39,4903 |  | Mean | 28,1321 |  | Mean | 33,8293 |
| Standard Deviation | 25,10328 |  | Standard Deviation | 24,54176 |  | Standard Deviation | 24,85335 |
| Percentile 25 | 25,0000 |  | Percentile 25 | 0,0000 |  | Percentile 25 | 16,6667 |
| Percentile 50 | 41,6667 |  | Percentile 50 | 25,0000 |  | Percentile 50 | 33,3333 |
| Percentile 75 | 58,3333 |  | Percentile 75 | 50,0000 |  | Percentile 75 | 50,0000 |
|  |  |  |  |  |  |  |  |
|  | |  |  | |  |  | |
|  | q18 |  |  | q18 |  |  | q18 |
| Position | 19 |  | Position | 20 |  | Position | 20 |
| Label | Jeg tænker over, hvordan grisene har haft det, når jeg køber svinekød - Hvor enig eller uenig er du i følgende udsagn? |  | Label | Wenn ich Schweinefleisch kaufe, denke ich darüber nach, unter welchen Bedingungen das Tier gelebt hat - Wie sehr stimmen Sie mit folgenden Aussagen überein?? |  | Label | Jag tänker på hur grisarna har haft det när jag köper fläskkött - ] I vilken grad håller du med om följande påståenden? |
| Type | Numeric |  | Type | Numeric |  | Type | Numeric |
| Format | F1 |  | Format | F1 |  | Format | F1 |
| Measurement | Nominal |  | Measurement | Nominal |  | Measurement | Nominal |
| Role | Input |  | Role | Input |  | Role | Input |
| 1 | Helt uenig |  | 1 | Ich stimme mit der Aussage ganz und gar nicht überein |  | 1 | Instämmer inte alls |
| 2 | Delvist uenig |  | 2 | Ich stimme teilweise nicht überein |  | 2 | Instämmer inte |
| 3 | Hverken eller |  | 3 | Weder noch |  | 3 | Varken eller |
| 4 | Delvist enig |  | 4 | Ich stimme teilweise überein |  | 4 | Instämmer delvis |
| 5 | Helt enig |  | 5 | Ich stimme mit der Aussage ganz und gar überein |  | 5 | Instämmer helt |
| 6 | Ved ikke |  | 6 | Weiß nicht |  | 6 | Vet inte |
| System |  |  | System |  |  | System |  |
|  |  |  |  |  |  |  |  |
|  | |  |  | |  |  | |
|  | q19 |  |  | q19 |  |  | q19 |
| Position | 20 |  | Position | 21 |  | Position | 21 |
| Label | Al den snak om dyrevelfærd er i mine øjne overdreven. - Hvor enig er du i de følgende spørgsmål, om dyrevelfærd? |  | Label | Die gesamte Tierwohldebatte ist in meinen Augen übertrieben.. - Wie sehr stimmen Sie mit folgenden Aussagen über das Tierwohl überein?? |  | Label | Allt prat om djurvälfärd är enligt mig överdrivet... - I vilken utsträckning instämmer du i följande frågor om djurvälfärd? |
| Type | Numeric |  | Type | Numeric |  | Type | Numeric |
| Format | F1 |  | Format | F1 |  | Format | F1 |
| Measurement | Nominal |  | Measurement | Nominal |  | Measurement | Nominal |
| Role | Input |  | Role | Input |  | Role | Input |
| 1 | Helt uenig |  | 1 | Ich stimme mit der Aussage ganz und gar nicht überein |  | 1 | Instämmer inte alls |
| 2 | Uenig |  | 2 | Lehne ich ab |  | 2 | Instämmer inte |
| 3 | Hverken enig eller uenig |  | 3 | Lehne ich weder ab noch stimme ich zu |  | 3 | Varken instämmer eller instämmer inte |
| 4 | Enig |  | 4 | Stimme zu |  | 4 | Instämmer |
| 5 | Helt enig |  | 5 | Ich stimme mit der Aussage ganz und gar überein |  | 5 | Instämmer helt |
| 6 | Ved ikke |  | 6 | Weiß nicht |  | 6 | Vet inte |
|  |  |  |  |  |  |  |  |
|  | |  |  | |  |  | |
|  | q20 |  |  | q20 |  |  | q20 |
| Position | 21 |  | Position | 22 |  | Position | 22 |
| Label | Samfundet har vigtigere ting, at beskæftige sig med end dyrs forhold. - Hvor enig er du i de følgende spørgsmål, om dyrevelfærd? |  | Label | Es gibt wichtigere gesellschaftliche Fragen als das Tierwohl - Wie sehr stimmen Sie mit folgenden Aussagen über das Tierwohl überein?? |  | Label | Samhället har viktigare saker att ägna sig åt än djurs förhållanden... - I vilken utsträckning instämmer du i följande frågor om djurvälfärd? |
| Type | Numeric |  | Type | Numeric |  | Type | Numeric |
| Format | F1 |  | Format | F1 |  | Format | F1 |
| Measurement | Nominal |  | Measurement | Nominal |  | Measurement | Nominal |
| Role | Input |  | Role | Input |  | Role | Input |
| 1 | Helt uenig |  | 1 | Ich stimme mit der Aussage ganz und gar nicht überein |  | 1 | Instämmer inte alls |
| 2 | Uenig |  | 2 | Lehne ich ab |  | 2 | Instämmer inte |
| 3 | Hverken enig eller uenig |  | 3 | Lehne ich weder ab noch stimme ich zu |  | 3 | Varken instämmer eller instämmer inte |
| 4 | Enig |  | 4 | Stimme zu |  | 4 | Instämmer |
| 5 | Helt enig |  | 5 | Ich stimme mit der Aussage ganz und gar überein |  | 5 | Instämmer helt |
| 6 | Ved ikke |  | 6 | Weiß nicht |  | 6 | Vet inte |
|  |  |  |  |  |  |  |  |
|  | |  |  | |  |  | |
|  | q21 |  |  | q21 |  |  | q21 |
| Position | 22 |  | Position | 23 |  | Position | 23 |
| Label | Jeg orker ikke at sætte mig ind i kravene til forskellige dyrevelfærdsmærker - Hvor enig eller uenig er du i følgende udsagn? |  | Label | Es überfordert mich, die Kriterien verschiedener Tierwohllabels zu verinnerlichen - Wie sehr stimmen Sie mit folgenden Aussagen überein?? |  | Label | Jag orkar inte sätta mig in i kraven för olika djurskyddsmärken - ] I vilken grad håller du med om följande påståenden? |
| Type | Numeric |  | Type | Numeric |  | Type | Numeric |
| Format | F1 |  | Format | F1 |  | Format | F1 |
| Measurement | Nominal |  | Measurement | Nominal |  | Measurement | Nominal |
| Role | Input |  | Role | Input |  | Role | Input |
| 1 | Helt uenig |  | 1 | Ich stimme mit der Aussage ganz und gar nicht überein |  | 1 | Instämmer inte alls |
| 2 | Delvist uenig |  | 2 | Ich stimme teilweise nicht überein |  | 2 | Instämmer inte |
| 3 | Hverken eller |  | 3 | Weder noch |  | 3 | Varken eller |
| 4 | Delvist enig |  | 4 | Ich stimme teilweise überein |  | 4 | Instämmer delvis |
| 5 | Helt enig |  | 5 | Ich stimme mit der Aussage ganz und gar überein |  | 5 | Instämmer helt |
| 6 | Ved ikke |  | 6 | Weiß nicht |  | 6 | Vet inte |
|  |  |  |  |  |  |  |  |
|  | |  |  | |  |  | |
|  | q22 |  |  | q22 |  |  | q22 |
| Position | 23 |  | Position | 24 |  | Position | 24 |
| Label | Attitude strength |  | Label | <none> |  | Label | <none> |
| Type | Numeric |  | Type | Numeric |  | Type | Numeric |
| Format | F8.2 |  | Format | F8.2 |  | Format | F8.2 |
| Measurement | Nominal |  | Measurement | Nominal |  | Measurement | Nominal |
| Role | Input |  | Role | Input |  | Role | Input |
| ,00 |  |  | ,00 |  |  | ,00 |  |
| 1,00 |  |  | 1,00 |  |  | 1,00 |  |
| 2,00 |  |  | 2,00 |  |  | 2,00 |  |
| 3,00 |  |  | 3,00 |  |  | 3,00 |  |
| 4,00 |  |  | 4,00 |  |  | 4,00 |  |
| System |  |  | System |  |  | System |  |
|  |  |  |  |  |  |  |  |
|  | |  |  | |  |  | |
|  | q23 |  |  | q23 |  |  | q23 |
| Position | 24 |  | Position | 25 |  | Position | 25 |
| Label | Svinekød fra indendørs produktion mærket med fx Fødevarestyrelsens velfærdsmærke 'Bedre dyrevelfærd', COOP's dyrevelfærdshjerte eller Antonius - Hvor ofte køber du følgende slags svinekød? |  | Label | Schweinefleisch mit Labels wie Initiative Tierwohl, Haltungsform, Für Mehr Tierschutz, Tierschutz kontrolliert oder Neuland - Wie oft kaufen Sie folgende Zubereitungsarten?? |  | Label | Ekologiskt fläskkött - Hur ofta köper du följande sorters fläskkött? |
| Type | Numeric |  | Type | Numeric |  | Type | Numeric |
| Format | F1 |  | Format | F1 |  | Format | F1 |
| Measurement | Nominal |  | Measurement | Nominal |  | Measurement | Nominal |
| Role | Input |  | Role | Input |  | Role | Input |
| 1 | Aldrig |  | 1 | Niemals |  | 1 | Aldrig |
| 2 | Sjældent |  | 2 | Selten |  | 2 | Sällan |
| 3 | Nogle gange |  | 3 | Gelegentlich |  | 3 | Ibland |
| 4 | Ofte |  | 4 | Häufig |  | 4 | Ofta |
| 5 | Stort set hver gang jeg køber svinekød |  | 5 | Fast immer, wenn ich Schweinefleisch kaufe |  | 5 | Nästan varje gång jag köper fläskkött |
| 6 | Ved ikke |  | 6 | Weiß nicht |  | 6 | Vet inte |
| System |  |  | System |  |  | System |  |
|  |  |  |  |  |  |  |  |
|  | |  |  | |  |  | |
|  | q24 |  |  | q24 |  |  | q24 |
| Position | 25 |  | Position | 26 |  | Position | 26 |
| Label | Svinekød fra udendørs produktion mærket med fx Økologisk Svinekød, Friland Svinekød eller 'Anbefalet af Dyrenes Beskyttelse' - Hvor ofte køber du følgende slags svinekød? |  | Label | Bio-Schweinefleisch (z. B. Naturland) - Wie oft kaufen Sie folgende Zubereitungsarten?? |  | Label | Vanligt fläskkött - Hur ofta köper du följande sorters fläskkött? |
| Type | Numeric |  | Type | Numeric |  | Type | Numeric |
| Format | F1 |  | Format | F1 |  | Format | F1 |
| Measurement | Nominal |  | Measurement | Nominal |  | Measurement | Nominal |
| Role | Input |  | Role | Input |  | Role | Input |
| 1 | Aldrig |  | 1 | Niemals |  | 1 | Aldrig |
| 2 | Sjældent |  | 2 | Selten |  | 2 | Sällan |
| 3 | Nogle gange |  | 3 | Gelegentlich |  | 3 | Ibland |
| 4 | Ofte |  | 4 | Häufig |  | 4 | Ofta |
| 5 | Stort set hver gang jeg køber svinekød |  | 5 | Fast immer, wenn ich Schweinefleisch kaufe |  | 5 | Nästan varje gång jag köper fläskkött |
| 6 | Ved ikke |  | 6 | Weiß nicht |  | 6 | Vet inte |
| System |  |  | System |  |  | System |  |
|  |  |  |  |  |  |  |  |
|  | |  |  | |  |  | |
|  | q25 |  |  | q25 |  |  |  |
| Position | 26 |  | Position | 27 |  |  |  |
| Label | Konventionelt/almindeligt svinekød - Hvor ofte køber du følgende slags svinekød? |  | Label | Konventionelles/herkömmliches Schweinefleisch - Wie oft kaufen Sie folgende Zubereitungsarten?? |  |  |  |
| Type | Numeric |  | Type | Numeric |  |  |  |
| Format | F1 |  | Format | F1 |  |  |  |
| Measurement | Nominal |  | Measurement | Nominal |  |  |  |
| Role | Input |  | Role | Input |  |  |  |
| 1 | Aldrig |  | 1 | Niemals |  |  |  |
| 2 | Sjældent |  | 2 | Selten |  |  |  |
| 3 | Nogle gange |  | 3 | Gelegentlich |  |  |  |
| 4 | Ofte |  | 4 | Häufig |  |  |  |
| 5 | Stort set hver gang jeg køber svinekød |  | 5 | Fast immer, wenn ich Schweinefleisch kaufe |  |  | |
| 6 | Ved ikke |  | 6 | Weiß nicht |  |  |  |
| System |  |  | System |  |  |  |  |
|  |  |  |  |  |  |  |  |
|  | |  |  | |  |  |  |
|  | q26 |  |  | q26 |  |  |  |
| Position | 27 |  | Position | 28 |  |  |  |
| Label | WFPorkType1 |  | Label | <none> |  |  |  |
| Type | Numeric |  | Type | Numeric |  |  |  |
| Format | F8.2 |  | Format | F8.2 |  |  |  |
| Measurement | Nominal |  | Measurement | Nominal |  |  |  |
| Role | Input |  | Role | Input |  |  |  |
| ,00 |  |  | ,00 |  |  |  |  |
| 1,00 |  |  | 1,00 |  |  |  |  |
| System |  |  | System |  |  |  |  |
|  |  |  |  |  |  |  |  |
|  | |  |  | |  |  |  |
|  | q27 |  |  | q27 |  |  | q25 |
| Position | 28 |  | Position | 29 |  | Position | 27 |
| Label | WFPorkType2 |  | Label | WFPorkType1 |  | Label | WFPorkType2 |
| Type | Numeric |  | Type | Numeric |  | Type | Numeric |
| Format | F8.2 |  | Format | F8.2 |  | Format | F8.2 |
| Measurement | Nominal |  | Measurement | Nominal |  | Measurement | Nominal |
| Role | Input |  | Role | Input |  | Role | Input |
| ,00 |  |  | ,00 |  |  | ,00 |  |
| 1,00 |  |  | 1,00 |  |  | 1,00 |  |
| System |  |  | System |  |  | System |  |
|  |  |  |  |  |  |  |  |
|  | |  |  | |  |  | |
|  | q28 |  |  | q28 |  |  | q26 |
| Position | 29 |  | Position | 30 |  | Position | 28 |
| Label | ConventionalPork |  | Label | ConventionalPork |  | Label | ConventionalPork |
| Type | Numeric |  | Type | Numeric |  | Type | Numeric |
| Format | F8.2 |  | Format | F8.2 |  | Format | F8.2 |
| Measurement | Nominal |  | Measurement | Nominal |  | Measurement | Nominal |
| Role | Input |  | Role | Input |  | Role | Input |
| ,00 |  |  | ,00 |  |  | ,00 |  |
| 1,00 |  |  | 1,00 |  |  | 1,00 |  |
| System |  |  | System |  |  | System |  |
|  |  |  |  |  |  |  |  |
|  | |  |  | |  |  |  |
|  | q29 |  |  | q29 |  |  | q27 |
| Position | 30 |  | Position | 31 |  | Position | 29 |
| Label | Er du mand eller kvinde? |  | Label | Welches Geschlecht haben Sie? |  | Label | Är du man eller kvinna? |
| Type | Numeric |  | Type | Numeric |  | Type | Numeric |
| Format | F1 |  | Format | F1 |  | Format | F1 |
| Measurement | Nominal |  | Measurement | Nominal |  | Measurement | Nominal |
| Role | Input |  | Role | Input |  | Role | Input |
| 1 | Mand |  | 1 | Male |  | 1 | Man |
| 2 | Kvinde |  | 2 | Female |  | 2 | Kvinna |
|  |  |  |  |  |  |  |  |
|  | |  |  | |  |  |  |
|  | q30 |  |  | q30 |  |  | q28 |
| Position | 31 |  | Position | 32 |  | Position | 30 |
| Label | Hvad er din alder? |  | Label | Wie alt sind Sie ? |  | Label | Hur gammal är du? |
| Type | Numeric |  | Type | Numeric |  | Type | Numeric |
| Format | F19 |  | Format | F19 |  | Format | F19 |
| Measurement | Nominal |  | Measurement | Nominal |  | Measurement | Nominal |
| Role | Input |  | Role | Input |  | Role | Input |
| 18 |  |  | 19 |  |  | 18 |  |
| 19 |  |  | 20 |  |  | 19 |  |
| 20 |  |  | 21 |  |  | 20 |  |
| 21 |  |  | 22 |  |  | 21 |  |
| 22 |  |  | 23 |  |  | 22 |  |
| 23 |  |  | 24 |  |  | 23 |  |
| 24 |  |  | 25 |  |  | 24 |  |
| 25 |  |  | 26 |  |  | 25 |  |
| 26 |  |  | 27 |  |  | 26 |  |
| 27 |  |  | 28 |  |  | 27 |  |
| 28 |  |  | 29 |  |  | 28 |  |
| 29 |  |  | 30 |  |  | 29 |  |
| 30 |  |  | 31 |  |  | 30 |  |
| 31 |  |  | 32 |  |  | 31 |  |
| 32 |  |  | 33 |  |  | 32 |  |
| 33 |  |  | 34 |  |  | 33 |  |
| 34 |  |  | 35 |  |  | 34 |  |
| 35 |  |  | 36 |  |  | 35 |  |
| 36 |  |  | 37 |  |  | 36 |  |
| 37 |  |  | 38 |  |  | 37 |  |
| 38 |  |  | 39 |  |  | 38 |  |
| 39 |  |  | 40 |  |  | 39 |  |
| 40 |  |  | 41 |  |  | 40 |  |
| 41 |  |  | 42 |  |  | 41 |  |
| 42 |  |  | 43 |  |  | 42 |  |
| 43 |  |  | 44 |  |  | 43 |  |
| 44 |  |  | 45 |  |  | 44 |  |
| 45 |  |  | 46 |  |  | 45 |  |
| 46 |  |  | 47 |  |  | 46 |  |
| 47 |  |  | 48 |  |  | 47 |  |
| 48 |  |  | 49 |  |  | 48 |  |
| 49 |  |  | 50 |  |  | 49 |  |
| 50 |  |  | 51 |  |  | 50 |  |
| 51 |  |  | 52 |  |  | 51 |  |
| 52 |  |  | 53 |  |  | 52 |  |
| 53 |  |  | 54 |  |  | 53 |  |
| 54 |  |  | 55 |  |  | 54 |  |
| 55 |  |  | 56 |  |  | 55 |  |
| 56 |  |  | 57 |  |  | 56 |  |
| 57 |  |  | 58 |  |  | 57 |  |
| 58 |  |  | 59 |  |  | 58 |  |
| 59 |  |  | 60 |  |  | 59 |  |
| 60 |  |  | 61 |  |  | 60 |  |
| 61 |  |  | 62 |  |  | 61 |  |
| 62 |  |  | 63 |  |  | 62 |  |
| 63 |  |  | 64 |  |  | 63 |  |
| 64 |  |  | 65 |  |  | 64 |  |
| 65 |  |  | 66 |  |  | 65 |  |
| 66 |  |  | 67 |  |  | 66 |  |
| 67 |  |  | 68 |  |  | 67 |  |
| 68 |  |  | 69 |  |  | 68 |  |
| 69 |  |  | 70 |  |  | 69 |  |
| 70 |  |  | 71 |  |  | 70 |  |
| 71 |  |  | 72 |  |  | 71 |  |
| 72 |  |  | 73 |  |  | 72 |  |
| 73 |  |  | 74 |  |  | 73 |  |
| 74 |  |  | 75 |  |  | 74 |  |
| 75 |  |  | 76 |  |  | 75 |  |
| 76 |  |  | 77 |  |  | 76 |  |
| 77 |  |  | 78 |  |  | 77 |  |
| 78 |  |  | 79 |  |  | 78 |  |
| 79 |  |  | 80 |  |  | 79 |  |
| 80 |  |  | 81 |  |  | 80 |  |
| 81 |  |  | 82 |  |  | 81 |  |
| 82 |  |  | 86 |  |  | 82 |  |
| 83 |  |  | 87 |  |  | 83 |  |
| 84 |  |  |  |  |  | 84 |  |
| 85 |  |  |  |  |  | 85 |  |
| 86 |  |  |  |  |  | 87 |  |
| 87 |  |  |  |  |  | 89 |  |
| 89 |  |  |  |  |  |  |  |
| 90 |  |  |  |  |  |  |  |
| 97 |  |  |  |  |  |  |  |
|  |  |  |  |  | |  |  |
|  | |  |  |  |  |  | |
|  | q31 |  |  | q31 |  |  | q29 |
| Position | 32 |  | Position | 33 |  | Position | 31 |
| Label | <none> |  | Label | <none> |  | Label | <none> |
| Type | Numeric |  | Type | Numeric |  | Type | Numeric |
| Format | F8.2 |  | Format | F8.2 |  | Format | F8.2 |
| Measurement | Nominal |  | Measurement | Nominal |  | Measurement | Nominal |
| Role | Input |  | Role | Input |  | Role | Input |
| 1,00 | 0-300000 DKR/year |  | 1,00 | 0-1500 Euro/monat |  | 1,00 | 0-300000 SEK/year |
| 2,00 | 300-500000 DKR/year |  | 2,00 | 15-2500 Euro/monat |  | 2,00 | 300-500000 SEK/year |
| 3,00 | 500-700000 DKR/year |  | 3,00 | 25-3500 Euro/monat |  | 3,00 | 500-700000 SEK/year |
| 4,00 | >700000 DKR/year |  | 4,00 | >3500 Euro/monat |  | 4,00 | >700000 SEK/year |
| 5,00 | Don´t know/won´t tell |  | 5,00 | Don´t know/won´t tell |  | 5,00 | Don´t know/won´t tell |
|  |  |  |  |  |  |  |  |
|  | |  |  | |  |  | |
|  | q32 |  |  | q32 |  |  | q30 |
| Position | 33 |  | Position | 34 |  | Position | 32 |
| Label | At jeg kan få det i den butik, jeg normalt handler i - Hvad er vigtigt for dig, når du køber svinekød? |  | Label | Dass ich es in dem Laden bekommen kann, in dem ich normalerweise einkaufe - Was ist Ihnen beim Schweinefleisch-Kauf am wichtigsten? |  | Label | Att jag kan köpa det i mataffären där jag vanligtvis handlar - Vad är viktigt för dig när du köper fläskkött? |
| Type | Numeric |  | Type | Numeric |  | Type | Numeric |
| Format | F1 |  | Format | F1 |  | Format | F1 |
| Measurement | Nominal |  | Measurement | Nominal |  | Measurement | Nominal |
| Role | Input |  | Role | Input |  | Role | Input |
| 0 | Not selected |  | 0 | Not selected |  | 0 | Not selected |
| 1 | At jeg kan få det i den butik, jeg normalt handler i |  | 1 | Dass ich es in dem Laden bekommen kann, in dem ich normalerweise einkaufe |  | 1 | Att jag kan köpa det i mataffären där jag vanligtvis handlar |
| System |  |  | System |  |  | System |  |
|  |  |  |  |  |  |  |  |
|  | |  |  | |  |  | |
|  | q33 |  |  | q33 |  |  | q31 |
| Position | 34 |  | Position | 35 |  | Position | 33 |
| Label | Passende udskæringer og pakkestørrelser - Hvad er vigtigt for dig, når du køber svinekød? |  | Label | Passende Portions- und Verpackungsgrößen - Was ist Ihnen beim Schweinefleisch-Kauf am wichtigsten? |  | Label | Passande stycken och förpackningsstorlekar - Vad är viktigt för dig när du köper fläskkött? |
| Type | Numeric |  | Type | Numeric |  | Type | Numeric |
| Format | F1 |  | Format | F1 |  | Format | F1 |
| Measurement | Nominal |  | Measurement | Nominal |  | Measurement | Nominal |
| Role | Input |  | Role | Input |  | Role | Input |
| 0 | Not selected |  | 0 | Not selected |  | 0 | Not selected |
| 1 | Passende udskæringer og pakkestørrelser |  | 1 | Passende Portions- und Verpackungsgrößen |  | 1 | Passande stycken och förpackningsstorlekar |
| System |  |  | System |  |  | System |  |
|  |  |  |  |  |  |  |  |
|  | |  |  | |  |  | |
|  | q34 |  |  | q34 |  |  | q32 |
| Position | 35 |  | Position | 36 |  | Position | 34 |
| Label | Dansk produktion - Hvad er vigtigt for dig, når du køber svinekød? |  | Label | Fleisch aus deutscher Produktion - Was ist Ihnen beim Schweinefleisch-Kauf am wichtigsten? |  | Label | Svensk produktion - Vad är viktigt för dig när du köper fläskkött? |
| Type | Numeric |  | Type | Numeric |  | Type | Numeric |
| Format | F1 |  | Format | F1 |  | Format | F1 |
| Measurement | Nominal |  | Measurement | Nominal |  | Measurement | Nominal |
| Role | Input |  | Role | Input |  | Role | Input |
| 0 | Not selected |  | 0 | Not selected |  | 0 | Not selected |
| 1 | Dansk produktion |  | 1 | Fleisch aus deutscher Produktion |  | 1 | Svensk produktion |
| System |  |  | System |  |  | System |  |
|  |  |  |  |  |  |  |  |
|  | |  |  | |  |  | |
|  | q35 |  |  | q35 |  |  | q33 |
| Position | 36 |  | Position | 37 |  | Position | 35 |
| Label | God smag - Hvad er vigtigt for dig, når du køber svinekød? |  | Label | Guter Geschmack - Was ist Ihnen beim Schweinefleisch-Kauf am wichtigsten? |  | Label | God smak - Vad är viktigt för dig när du köper fläskkött? |
| Type | Numeric |  | Type | Numeric |  | Type | Numeric |
| Format | F1 |  | Format | F1 |  | Format | F1 |
| Measurement | Nominal |  | Measurement | Nominal |  | Measurement | Nominal |
| Role | Input |  | Role | Input |  | Role | Input |
| 0 | Not selected |  | 0 | Not selected |  | 0 | Not selected |
| 1 | God smag |  | 1 | Guter Geschmack |  | 1 | God smak |
| System |  |  | System |  |  | System |  |
|  |  |  |  |  |  |  |  |
|  | |  |  | |  |  | |
|  | q36 |  |  | q36 |  |  | q34 |
| Position | 37 |  | Position | 38 |  | Position | 36 |
| Label | Billigt - Hvad er vigtigt for dig, når du køber svinekød? |  | Label | Preiswerte Angebote - Was ist Ihnen beim Schweinefleisch-Kauf am wichtigsten? |  | Label | Billigt - Vad är viktigt för dig när du köper fläskkött? |
| Type | Numeric |  | Type | Numeric |  | Type | Numeric |
| Format | F1 |  | Format | F1 |  | Format | F1 |
| Measurement | Nominal |  | Measurement | Nominal |  | Measurement | Nominal |
| Role | Input |  | Role | Input |  | Role | Input |
| 0 | Not selected |  | 0 | Not selected |  | 0 | Not selected |
| 1 | Billigt |  | 1 | Preiswerte Angebote |  | 1 | Billigt |
| System |  |  | System |  |  | System |  |
|  |  |  |  |  |  |  |  |
|  | |  |  | |  |  | |
|  | q37 |  |  | q37 |  |  | q35 |
| Position | 38 |  | Position | 39 |  | Position | 37 |
| Label | Lavt fedtindhold - Hvad er vigtigt for dig, når du køber svinekød? |  | Label | Niedriger Fettanteil - Was ist Ihnen beim Schweinefleisch-Kauf am wichtigsten? |  | Label | Lågt fettinnehåll - Vad är viktigt för dig när du köper fläskkött? |
| Type | Numeric |  | Type | Numeric |  | Type | Numeric |
| Format | F1 |  | Format | F1 |  | Format | F1 |
| Measurement | Nominal |  | Measurement | Nominal |  | Measurement | Nominal |
| Role | Input |  | Role | Input |  | Role | Input |
| 0 | Not selected |  | 0 | Not selected |  | 0 | Not selected |
| 1 | Lavt fedtindhold |  | 1 | Niedriger Fettanteil |  | 1 | Lågt fettinnehåll |
| System |  |  | System |  |  | System |  |
|  |  |  |  |  |  |  |  |
|  | |  |  | |  |  | |
|  | q38 |  |  | q38 |  |  | q36 |
| Position | 39 |  | Position | 40 |  | Position | 38 |
| Label | Lav miljøpåvirkning (fx lav forurening af søer og vandløb) - Hvad er vigtigt for dig, når du køber svinekød? |  | Label | Geringe Umweltbelastung (z. B. niedrige Belastung von Gewässern) - Was ist Ihnen beim Schweinefleisch-Kauf am wichtigsten? |  | Label | Låg miljöpåverkan (t.ex. låg förorening av sjöar och vattendrag)Lav miljøpåvirkning (fx lav forurening af søer og vandløb) - Vad är viktigt för dig när du köper fläskkött? |
| Type | Numeric |  | Type | Numeric |  | Type | Numeric |
| Format | F1 |  | Format | F1 |  | Format | F1 |
| Measurement | Nominal |  | Measurement | Nominal |  | Measurement | Nominal |
| Role | Input |  | Role | Input |  | Role | Input |
| 0 | Not selected |  | 0 | Not selected |  | 0 | Not selected |
| 1 | Lav miljøpåvirkning (fx lav forurening af søer og vandløb) |  | 1 | Geringe Umweltbelastung (z. B. niedrige Belastung von Gewässern) |  | 1 | Låg miljöpåverkan (t.ex. låg förorening av sjöar och vattendrag)Lav miljøpåvirkning (fx lav forurening af søer og vandlø |
| System |  |  | System |  |  | System |  |
|  |  |  |  |  |  |  |  |
|  | |  |  | |  |  | |
|  | q39 |  |  | q39 |  |  | q37 |
| Position | 40 |  | Position | 41 |  | Position | 39 |
| Label | Lav klimapåvirkning - Hvad er vigtigt for dig, når du køber svinekød? |  | Label | Geringer Klimaeinfluss - Was ist Ihnen beim Schweinefleisch-Kauf am wichtigsten? |  | Label | Låg klimatpåverkan - Vad är viktigt för dig när du köper fläskkött? |
| Type | Numeric |  | Type | Numeric |  | Type | Numeric |
| Format | F1 |  | Format | F1 |  | Format | F1 |
| Measurement | Nominal |  | Measurement | Nominal |  | Measurement | Nominal |
| Role | Input |  | Role | Input |  | Role | Input |
| 0 | Not selected |  | 0 | Not selected |  | 0 | Not selected |
| 1 | Lav klimapåvirkning |  | 1 | Geringer Klimaeinfluss |  | 1 | Låg klimatpåverkan |
| System |  |  | System |  |  | System |  |
|  |  |  |  |  |  |  |  |
|  | |  |  | |  |  | |
|  | q40 |  |  | q40 |  |  | q38 |
| Position | 41 |  | Position | 42 |  | Position | 40 |
| Label | Høj fødevaresikkerhed (ingen bakterier i kødet) - Hvad er vigtigt for dig, når du køber svinekød? |  | Label | Lebensmittelsicherheit (keine bakterielle Belastung) - Was ist Ihnen beim Schweinefleisch-Kauf am wichtigsten? |  | Label | Hög livsmedelssäkerhet (inga bakterier i köttet) - Vad är viktigt för dig när du köper fläskkött? |
| Type | Numeric |  | Type | Numeric |  | Type | Numeric |
| Format | F1 |  | Format | F1 |  | Format | F1 |
| Measurement | Nominal |  | Measurement | Nominal |  | Measurement | Nominal |
| Role | Input |  | Role | Input |  | Role | Input |
| 0 | Not selected |  | 0 | Not selected |  | 0 | Not selected |
| 1 | Høj fødevaresikkerhed (ingen bakterier i kødet) |  | 1 | Lebensmittelsicherheit (keine bakterielle Belastung) |  | 1 | Hög livsmedelssäkerhet (inga bakterier i köttet) |
| System |  |  | System |  |  | System |  |
|  |  |  |  |  |  |  |  |
|  | |  |  | |  |  | |
|  | q41 |  |  | q41 |  |  | q39 |
| Position | 42 |  | Position | 43 |  | Position | 41 |
| Label | Kontrol med, at regler overholdes - Hvad er vigtigt for dig, når du køber svinekød? |  | Label | Kontrollierte Tierhaltung - Was ist Ihnen beim Schweinefleisch-Kauf am wichtigsten? |  | Label | Kontroll av att regler efterlevs - Vad är viktigt för dig när du köper fläskkött? |
| Type | Numeric |  | Type | Numeric |  | Type | Numeric |
| Format | F1 |  | Format | F1 |  | Format | F1 |
| Measurement | Nominal |  | Measurement | Nominal |  | Measurement | Nominal |
| Role | Input |  | Role | Input |  | Role | Input |
| 0 | Not selected |  | 0 | Not selected |  | 0 | Not selected |
| 1 | Kontrol med, at regler overholdes |  | 1 | Kontrollierte Tierhaltung |  | 1 | Kontroll av att regler efterlevs |
| System |  |  | System |  |  | System |  |
|  |  |  |  |  |  |  |  |
|  | |  |  | |  |  | |
|  | q42 |  |  | q42 |  |  | q40 |
| Position | 43 |  | Position | 44 |  | Position | 42 |
| Label | Lille brug af antibiotika - Hvad er vigtigt for dig, når du køber svinekød? |  | Label | Kleine Verwendung von Antibiotika - Was ist Ihnen beim Schweinefleisch-Kauf am wichtigsten? |  | Label | Låg användning av antibiotika - Vad är viktigt för dig när du köper fläskkött? |
| Type | Numeric |  | Type | Numeric |  | Type | Numeric |
| Format | F1 |  | Format | F1 |  | Format | F1 |
| Measurement | Nominal |  | Measurement | Nominal |  | Measurement | Nominal |
| Role | Input |  | Role | Input |  | Role | Input |
| 0 | Not selected |  | 0 | Not selected |  | 0 | Not selected |
| 1 | Lille brug af antibiotika |  | 1 | Kleine Verwendung von Antibiotika |  | 1 | Låg användning av antibiotika |
| System |  |  | System |  |  | System |  |
|  |  |  |  |  |  |  |  |
|  | |  |  | |  |  | |
|  | q43 |  |  | q43 |  |  | q41 |
| Position | 44 |  | Position | 45 |  | Position | 43 |
| Label | Ingen GMO-foder - Hvad er vigtigt for dig, når du køber svinekød? |  | Label | Kein gentechnisch modifiziertes Tierfutter - Was ist Ihnen beim Schweinefleisch-Kauf am wichtigsten? |  | Label | Inget GMO-foder - Vad är viktigt för dig när du köper fläskkött? |
| Type | Numeric |  | Type | Numeric |  | Type | Numeric |
| Format | F1 |  | Format | F1 |  | Format | F1 |
| Measurement | Nominal |  | Measurement | Nominal |  | Measurement | Nominal |
| Role | Input |  | Role | Input |  | Role | Input |
| 0 | Not selected |  | 0 | Not selected |  | 0 | Not selected |
| 1 | Ingen GMO-foder |  | 1 | Kein gentechnisch modifiziertes Tierfutter |  | 1 | Inget GMO-foder |
| System |  |  | System |  |  | System |  |
|  |  |  |  |  |  |  |  |
|  | |  |  | |  |  |  |
|  | q44 |  |  | q44 |  |  | q44 |
| Weight | 1612 |  | Weight | 1607 |  | Weight | 1613 |
| Valid N (listwise) | 1612 |  | Valid N (listwise) | 1607 |  | Valid N (listwise) | 1613 |
|  | Mean |  |  | Mean |  |  | Mean |
|  | 1,0000 |  |  | 1,000000 |  |  | 1,000000 |
|  | Minimum |  |  | Minimum |  |  | Minimum |
|  | 0,31 |  |  | 0,3123 |  |  | 0,3593 |
|  | Maximum |  |  | Maximum |  |  | Maximum |
|  | 6,00 |  |  | 8,4175 |  |  | 3,4730 |
|  | Std. Deviation |  |  | Std. Deviation |  |  | Std. Deviation |
|  | 0,98043 |  |  | 0,9934142 |  |  | 0,4708557 |
