# Supplementary material for: Moral Convictions and Meat Consumption—A Comparative Study of the Animal Ethics Orientations of Consumers of Pork in Denmark, Germany, and Sweden
Source: Animals (Basel). 2021 Jan 28;11(2):329. doi: 10.3390/ani11020329 (PMC7912257; doi:10.3390/ani11020329)
Supplement: Supplementary file 1 [file animals-11-00329-s001.zip › supple/Supplementary File 1.docx]

**Supplementary material 1**

| **Table S1.** Language-specific versions of the animal ethics questionnaire statements | | | | |
| --- | --- | --- | --- | --- |
| **Animal ethics orientation question belongs to** | **English question wording** | **Danish question wording** | **German question wording** | **Swedish question wording** |
| Animal rights | The use of animals by humans should be prohibited by law | Det burde forbydes ved lov, at mennesker bruger dyr. | Die Nutzung von Tieren sollte gesetzlich verboten werden. | Det bör vara förbjudet enligt lag för människor att använda djur. |
|  | In principle, the use of animals by humans is unacceptable because animals can feel pain. happiness. etc. | Det er som udgangspunkt uacceptabelt at mennesker bruger dyr, fordi dyr kan føle smerte, glæde og lignende. | Grundsätzlich ist es inakzeptabel, dass der Mensch Tiere nutzt, da diese Schmerzen, Freude usw. empfinden. | Det är generellt sett oacceptabelt att människor använder djur eftersom djur kan känna smärta, glädje och liknande. |
|  | In principle, the use of animals by humans is unacceptable because animals are sentient beings. | Det er som udgangspunkt uacceptabelt at mennesker bruger dyr, fordi dyr er væsner, som sanser og tænker. | Grundsätzlich ist es inakzeptabel, dass der Mensch Tiere nutzt, da Tiere Wesen sind, die fühlen und denken. | Det är generellt sett oacceptabelt att människor använder djur eftersom djur är varelser som känner och tänker. |
| Anthro-pocentric | We have the right to use animals because humans are intellectually superior to animals. | Vi har ret til at bruge dyr, fordi mennesker er intellektuelt overlegne i forhold til dyrene. | Wir haben das Recht, Tiere zu unseren Zwecken zu nutzen, da wir intellektuell überlegen sind. | Vi har rätt att använda djur eftersom människor är intellektuellt överlägsna djuren. |
|  | Human interests are more important than those of animals. | Menneskehedens interesser er vigtigere end dyrs interesser. | Die Interessen des Menschen sind wichtiger als die Interessen der Tiere. | Mänsklighetens intressen är viktigare än djurens intressen. |
|  | We must prioritize humans over animals. | Vi skal tage hensyn til mennesker frem for dyr. | Wir sollten mehr Rücksicht auf Menschen als auf Tiere nehmen. | Vi måste ta hänsyn till människor snarare än djur. |
| Animal protection | Using animals for important human purposes (e.g. medical research) is acceptable if it is done so that the animals do not experience unnecessary stress. | Det er acceptabelt at bruge dyr til vigtige menneskelige formål (f.eks. medicinsk forskning), hvis det sikres at dyrene ikke oplever unødig stress. | Die Nutzung von Tieren für wichtige menschliche Zielstellungen (wie z. B. der medizinischen Forschung) ist akzeptabel, wenn Tiere vor unnötigem Stress geschützt werden. | Det är acceptabelt att använda djur för viktiga mänskliga ändamål (t.ex. medicinsk forskning) om det säkerställs att djuren inte upplever otillbörlig stress. |
|  | Using animals for important human purposes is acceptable if it is done so that the animals do not experience unnecessary pain. | Det er acceptabelt at bruge dyr til vigtige menneskelige formål, hvis det sikres at dyrene ikke oplever unødig smerte og anden lidelse. | Die Nutzung von Tieren für wichtige menschliche Zielstellungen (wie z. B. der medizinischen Forschung) ist akzeptabel, wenn Tiere vor unnötigen Schmerzen und Leid geschützt werden. | Det är acceptabelt att använda djur för viktiga mänskliga ändamål om det säkerställs att djuren inte upplever onödig smärta och andra sjukdomar. |
|  | Using animals for important human purposes is acceptable if the animals have a decent quality of life. | Det er acceptabelt at bruge dyr til vigtige menneskelige formål, hvis dyrene har et ordentligt liv. | Die Nutzung von Tieren für wichtige menschliche Zielstellungen ist akzeptabel, wenn die Tiere unter ordentlichen Verhältnissen gehalten werden. | Det är acceptabelt att använda djur för viktiga mänskliga ändamål om djuren har ett ordentligt liv. |
| Lay utilitarian | Inflicting serious pain on animals is acceptable if it is necessary in order to achieve a vital human goal – e.g. in medical research. | Det kan være acceptabelt at påføre dyr alvorlig smerte, hvis det er nødvendigt for at opnå et vitalt menneskeligt formål (f.eks. medicinsk forskning). | Es kann gerechtfertigt sein, Tieren erheblichen Schmerz zuzuführen, wenn dies einem vitalen menschlichen Bedürfnis (z. B. medizinische Forschung) dient. | Det kan vara acceptabelt att orsaka allvarlig smärta på djur, om det är nödvändigt för att uppnå ett viktigt mänskligt syfte (t.ex. medicinsk forskning). |
|  | Inflicting considerable pain on animals is justified if the purpose is sufficiently important - e.g. medical research. | Det kan godt forsvares at påføre dyr væsentlig smerte, hvis formålet er vigtigt nok (f.eks. medicinsk forskning). | Es ist gerechtfertigt, Tieren Schmerz zuzuführen, wenn dies einem wichtigen Zweck (z. B. medizinische Forschung) dient. | Att djur upplever stress och försämrad välfärd kan försvaras om det tjänar ett syfte som är tillräckligt viktigt. |
|  | Exposing animals to stress and reducing their welfare is justified if the purpose is sufficiently important. | Det kan godt forsvares at dyr oplever stress og velfærdsforringelser, hvis det tjener et formål, som er vigtigt nok. | Es ist gerechtfertigt, wenn Tiere Stress erleiden oder das Tierwohl eingeschränkt wird, wenn dies einem wichtigen Zweck dient. | Att djur upplever stress och försämrad välfärd kan försvaras om det tjänar ett syfte som är tillräckligt viktigt. |
|  | **English response options** | **Danish response options** | **German response options** | **Swedish response options** |
|  | Completely disagree | Helt uenig | Ich stimme mit der Aussage ganz und gar nicht überein | Instämmer inte alls |
|  | Disagree | Uenig | Lehne ich ab | Instämmer inte |
|  | Neither disagree nor agree | Hverken uenig eller enig | Lehne ich weder ab noch stimme ich zu | Varken instämmer eller instämmer inte |
|  | Agree | Enig | Stimme zu | Instämmer |
|  | Completely agree | Helt enig | Ich stimme mit der Aussage ganz und gar überein | Instämmer helt |
